# Not all reference samples are equal in single-cell transcriptomics of human kidney tissue

**DOI:** 10.1172/jci.insight.204385

**Published:** 2026-05-19

**Authors:** Rajasree Menon, Paul L. Kimmel, Edgar A. Otto, Lalita Subramanian, Christopher L. O’Connor, Bradley Godfrey, Cathy Smith, Fadhl Alakwaa, Celine C. Berthier, Minnie M. Sarwal, E. Steve Woodle, Laura Pyle, Ye Ji Choi, Patricia Ladd, John R. Sedor, Sylvia E. Rosas, Sushrut S. Waikar, Abhijit S. Naik, Ricardo Melo Ferreira, Michael T. Eadon, Markus Bitzer, Petter Bjornstad, Jeffrey B. Hodgin, Matthias Kretzler, for the Kidney Precision Medicine Project (KPMP)

**Affiliations:** 1 Department of Computational Medicine and Bioinformatics, University of Michigan Medical School, Ann Arbor, Michigan, USA.; 2 Department of Medicine, School of Medicine and Health Sciences, George Washington University, Washington, DC, USA.; 3 Division of Nephrology, Department of Internal Medicine, University of Michigan Medical School, Ann Arbor, Michigan, USA.; 4 Division of Transplant Surgery, Department of Surgery, School of Medicine, University of California, San Francisco, California, USA.; 5 Department of Surgery, University of Cincinnati College of Medicine, Cincinnati, Ohio, USA.; 6 Departments of Pediatrics and Medicine, Division of Metabolism, Endocrinology and Nutrition, University of Washington, Seattle, Washington, USA.; 7 Department of Radiology, Division of Renal Diseases and Hypertension, University of Colorado Anschutz School of Medicine, Aurora, Colorado, USA.; 8 Medical Specialty and Lerner Research Institutes, Cleveland Clinic and Cleveland Clinic Lerner College of Medicine, Case Western Reserve University, Cleveland, Ohio, USA.; 9 Kidney and Hypertension unit, Joslin Diabetes Center and Harvard Medical School, Boston, Massachusetts, USA.; 10 Section of Nephrology, Department of Medicine, Boston Medical Center and Boston University Chobanian & Avedisian School of Medicine, Boston, Massachusetts, USA.; 11 Department of Medicine, Indiana University School of Medicine, Indianapolis, Indiana, USA.; 12 Department of Pathology, University of Michigan Medical School, Ann Arbor, Michigan, USA.; 13 KPMP is detailed in Supplemental Acknowledgments.

**Keywords:** Endocrinology, Nephrology, Diabetes, Molecular pathology, Transcriptomics

## Abstract

Identifying mechanisms of kidney disease commonly involves comparing diseased samples with healthy reference tissues; however, the effects of variability in tissue procurement, storage, and donor characteristics remain underexplored. In this study, we systematically evaluated 3 reference tissue types — tumor nephrectomy (TN), pretransplant biopsies from living donors (LD), and percutaneous biopsies from healthy control volunteers (HC) — to determine their impact on differential gene expression across 3 diabetic kidney disease states. We observed distinct injury markers, cell state proportions, and gene signatures associated with procurement method, sex, and donor age. Adjustment for these confounding factors significantly influenced pathway analysis results. Specifically, correcting for age and sex eliminated significant enrichment of IFN-γ response when comparing the diabetes mellitus–resilient group and HC group. Processes related to biological aging were enriched in older reference tissues, potentially confounding disease-specific interpretations. Importantly, TNF signaling via NF-κB remained enriched in LD and TN samples relative to HC, even after accounting for confounders. These results underscore the critical importance of selecting appropriate control tissues and rigorously adjusting for confounding variables to reliably discern the molecular mechanisms underlying kidney diseases.

## Abstract 2

Identifying disease processes from single-cell kidney transcriptomics requires comparing diseased tissue with reference samples presumed to be disease-free. This study shows that reference tissue type, procurement method, and donor age and sex can substantially influence these comparisons, underscoring the need to select appropriate controls and to rigorously adjust for key covariates to reliably resolve the molecular mechanisms underlying kidney disease.

## Introduction

The heterogeneous nature of kidney diseases has hindered our understanding of disease pathogenesis. Advances in genomics technologies have begun to unveil the molecular mechanisms underlying kidney diseases (1–4), yet these discoveries have relied heavily on comparisons between diseased and reference tissue. Given the challenges of obtaining disease-free kidney tissue from healthy humans, a wide variety of sources for reference tissue have been used. Moreover, factors such as sample procurement, preservation method, tissue processing technology, sex, age, and underlying physiological condition of the donor may affect reference tissues and corresponding gene expression profiles (5).

As molecular analysis tools continue to advance, an understanding of the advantages and limitations of different reference tissue sources has become critical in the context of comparative analyses. In this study, we compared single-cell gene expression data from 3 reference tissue sources: percutaneous kidney research biopsies from healthy controls (HC group), unaffected parts of tumor-nephrectomies (TN group), and perioperative living kidney donor biopsies (LD group).

**Figure 1:**
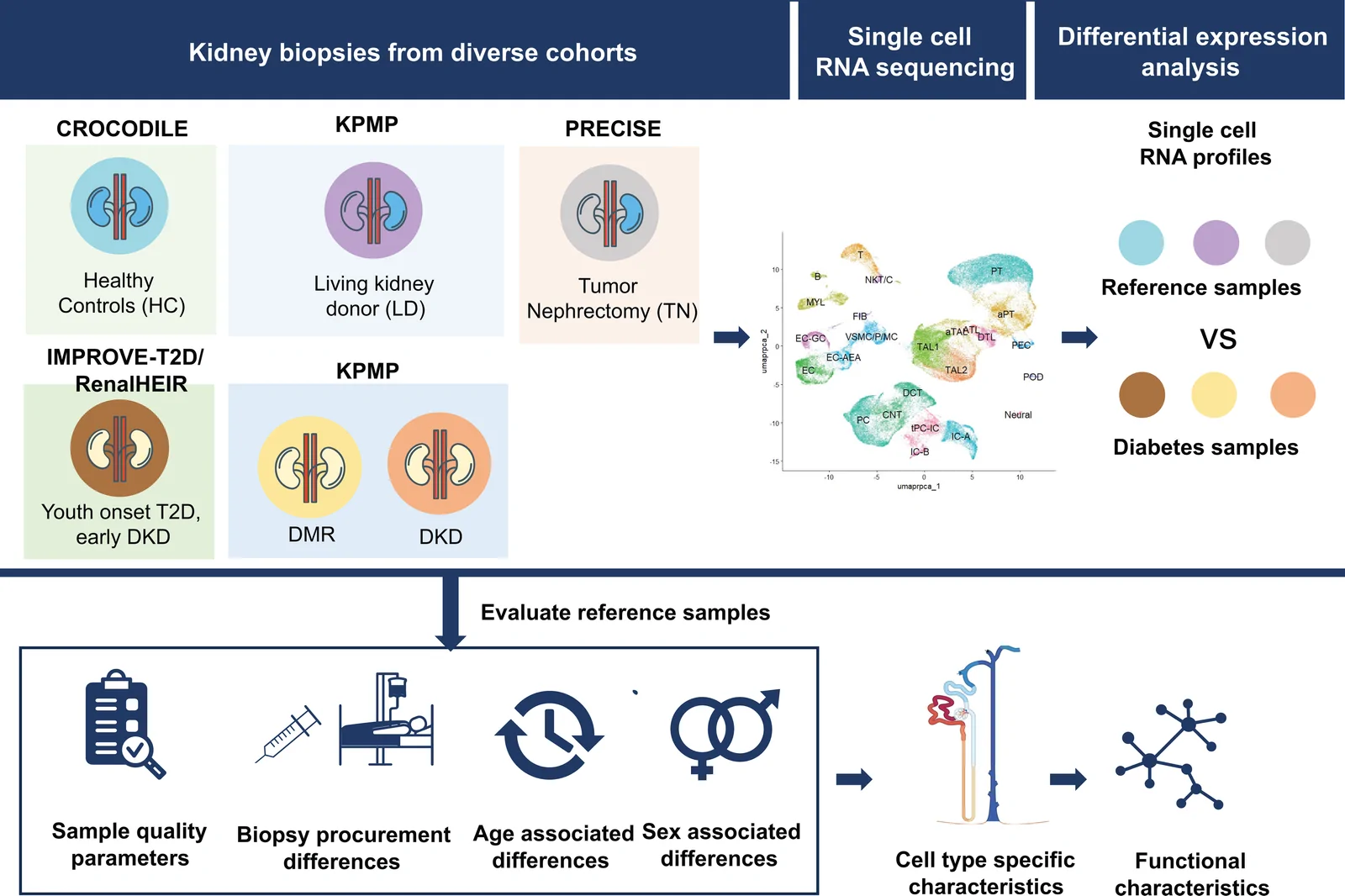
Overview of the analysis approach.

We analyzed single-cell gene expression data from percutaneous kidney research biopsies of patients with type 2 diabetes with diabetic kidney disease (DKD), patients with type 1 diabetes for over 25 years without clinical evidence of kidney disease (hereafter, diabetes mellitus resilient [DM-R]), and youth with early onset type 2 diabetes and kidney disease (early DKD) as disease comparators to determine how reference tissue selection affects identification of molecular mechanisms driving DKD progression (Figure 1).

## Results

**Figure 2:**
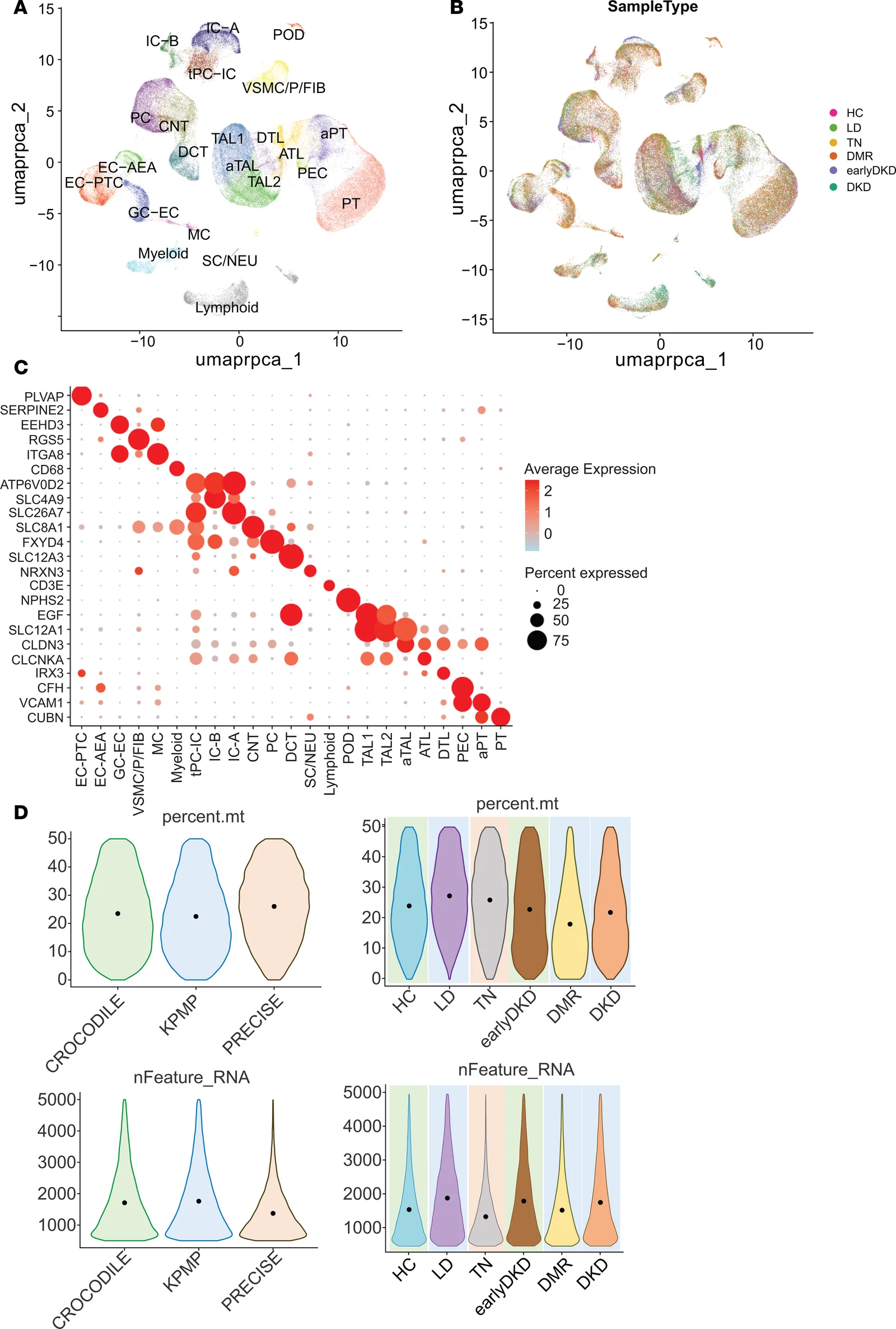
Single-cell data. (**A**) UMAP showing the annotated cell types from the integrated analysis of a total of 119,193 cells from 6 sample types: 20,000 cells each from LD (*n* = 9), TN (*n* = 9), DM-R (*n* = 18), early DKD (*n* = 9), and DKD (*n* = 17) and 19,193 cells from HC (*n* = 12). (**B**) UMAP of the integrated dataset showing successful integration of HC, LD, TN, DM-R, early DKD, and DKD. (**C**) Dot plot showing the specific markers for the annotated cell types. (**D**) Violin plots showing the quality control features: percentage of mitochondrial reads per cell (percent.mt) and number of features per cell (nFeature_RNA) in the 3 cohorts: CROCODILE/IMPROV-T2D, PRECISE, and KPMP and the 6 sample groups studied. The background color in the violin density plot of the sample groups indicates the cohort from which the samples were procured. POD, podocyte; PEC, parietal epithelial cell; PT, proximal tubule; aPT, adaptive/maladaptive; DTL, descending thin loop of Henle; ATL, ascending thin loop of Henle; TAL, thick ascending loop of Henle; aTAL, adaptive/maladaptive thick ascending loop of Henle; DCT, distal convoluted tubule; CNT, connecting tubule; PC, principal cell; IC-A, intercalated type A; IC-B, intercalated type B; tPC-IC, transient between PC and IC; EC, endothelial cell; EC-AEA, efferent and afferent arteriolar endothelial cells; EC-GC, glomerular endothelial cell; FIB, fibroblast; VSMC, vascular smooth muscle cell; P, pericyte; MC, mesangial cell; B, B cell; T, T cell; NK T/C, natural killer T cell.

*Single-cell and Visium data analysis*. In our down-sampled integrated single-cell dataset, comprising 119,193 cells from 74 samples across 6 sample groups, we annotated 23 distinct cell types (Figure 2A). Within these types, we identified 2 cell states for proximal tubule cells, healthy and adaptive/maladaptive (PT and aPT). The aPT state was specifically marked by overexpression of *VCAM1*, a known indicator of adaptive/maladaptive proximal cells (1). For the thick ascending loop of Henle cells (TAL), we identified 3 states: TAL1, TAL2, and aTAL, where the aTAL state was characterized by high expression of prominin 1 (*PROM1*) (1). Importantly, no batch effects were detected in the integrated dataset (Figure 2B, Supplemental Figure 1; supplemental material available online with this article; https://doi.org/10.1172/jci.insight.204385DS1). Supplemental Table 1 provides the number of cells per cell type identified from all the samples used in this study. Figure 2C shows the specific expression of markers used to annotate the cell types.

Single-cell analysis of kidney tissue, compared with other tissues, is known to have a high mitochondrial read content (6). In this study, we included only cells with 50% or fewer reads of the 13 protein-coding mitochondrial genes. The proportion of reads mapping to mitochondrial genes within a cell serves as an essential quality control metric and identifies stressed and damaged cells. Figure 2D presents violin plots of commonly used single-cell quality control features: mitochondrial read percentage per cell and number of genes per cell. Quality control metrics were largely comparable across the 6 sample groups representing the 3 cohorts (CROCODILE, KPMP, and PRECISE).

Uniform manifold approximation and projection (UMAP) visualization from the integrated analysis of 54 10X Genomics Visium datasets — including 13 DKD, 15 DM-R, 4 early DKD, 5 HC, 15 LD, and 2 TN samples — is presented in Supplemental Figure 2A. The merged dataset comprised 45,739 spots. Given that only DM-R, DKD, and LD groups had sufficient sample numbers, differential expression analyses with and without adjustment for confounders were conducted exclusively on these cohorts. Supplemental Figure 2B displays UMAPs for these 3 sample groups.

### Early stress and injury/disease markers

**Figure 3:**
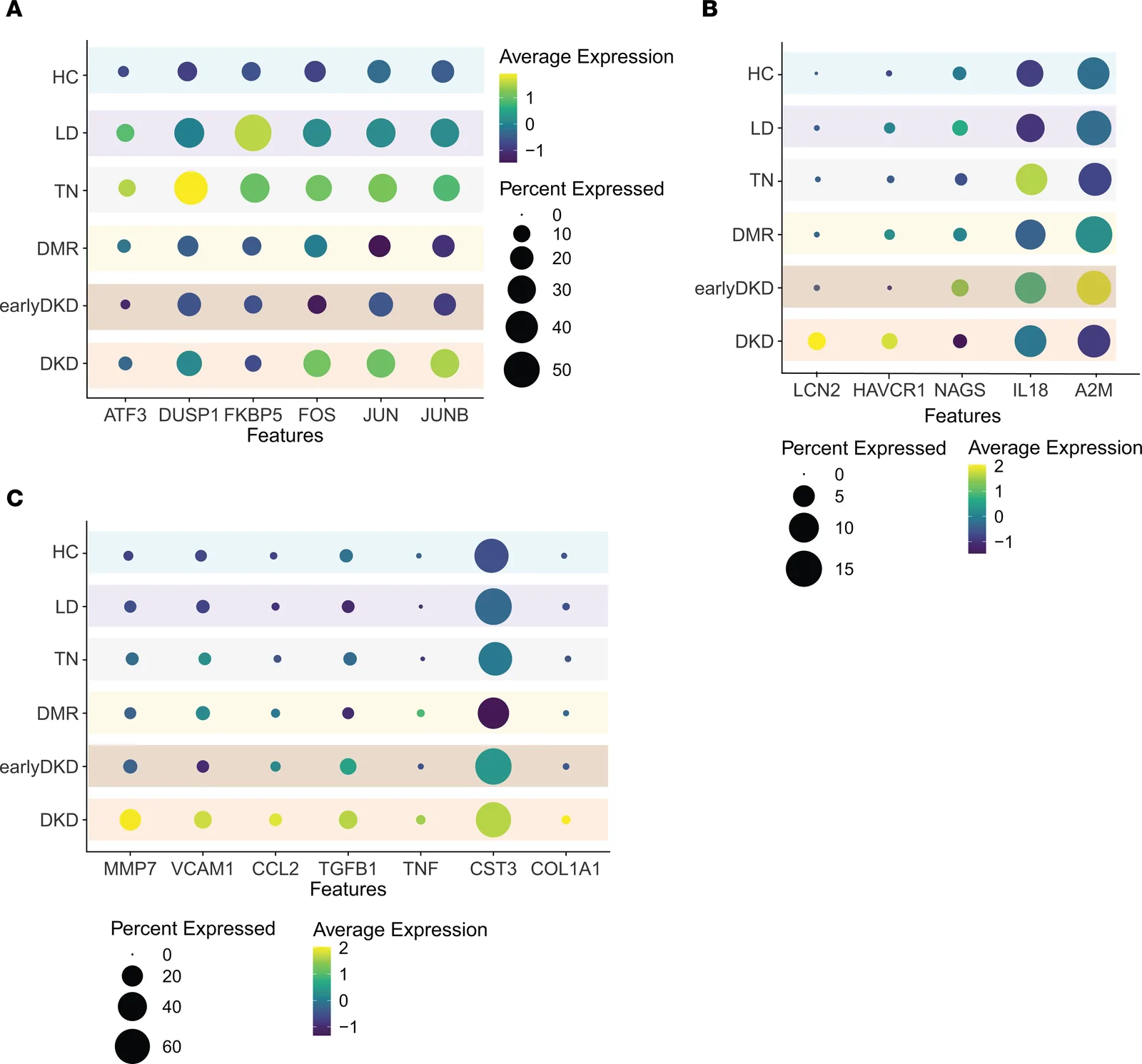
Early stress response and injury/disease markers. (**A**) Dot plot illustrating scaled expression of early stressor genes *ATF3*, *DUSP1*, *FKBP5*, *FOS*, *JUNB*, and *JUN* across 6 sample types. Expression was highest in TN and LD samples. (**B**) Dot plot showing expression of early CKD/injury markers in 6 sample types. Among reference groups, HC exhibited the lowest levels of *HAVCR1*, *LCN2*, and *IL18*. (**C**) Dot plot displaying expression of progressive/late disease markers across 6 sample types. Reference groups had relatively low expression compared with DKD, with little difference observed among reference groups.

Early stress response gene expression can be activated within minutes after stimulation (7, 8) and can serve as a sensitive biomarker of cellular stress. Activating transcription factor 3 (*ATF3*), dual specificity phosphatase 1 (*DUSP1*), FK506-binding protein 5 (*FKBP5*), fos proto-oncogene (*FOS*), *JUNB*, and *JUN* were lowest in HC and highest in TN among the reference tissues (Figure 3A). Both the LD and TN groups had higher expressions of these genes than the DM-R and early DKD groups.

In addition to the nonspecific stress markers studied above, kidney tissue–specific markers of cellular stress and damage have been developed and were assessed next. The expression levels of the following early kidney injury/disease markers are shown in Figure 3B: α-2 macroglobulin (*A2M*), hepatitis A virus cellular receptor 1 (*HAVCR1*, encoding kidney injury marker 1, KIM-1), IL-18 (*IL18*), lipocalin-2 (*LCN2*, encoding NGAL) and N-acetylglutamate synthase (*NAGS*). Among the reference groups, HC had the lowest expression for most of these genes. Although expressed at low levels, *HAVCR1* and *NAGS* markers of kidney damage were detected in greater percentage of cells with higher expression in LD compared with the other 2 reference groups (Figure 3B). *IL18*, an early indicator of acute kidney injury, showed relatively high expression levels in the TN group. No expression of *LCN2*, another early AKI marker, was observed in any of the reference groups.

Finally, chronic kidney disease (CKD) markers of chronic tissue stress and damage were evaluated. No differences in the late CKD markers, including CXCL2 (*CCL2*), collagen A1 (*COL1A1*), cystatin C (*CST3*), MMP7 (*MMP7*), TGF-β1 (*TGFB1*), TNF (*TNF*), and *VCAM1*, were observed among the reference tissue groups (Figure 3C).

#### Cell type proportion.

**Figure 4:**
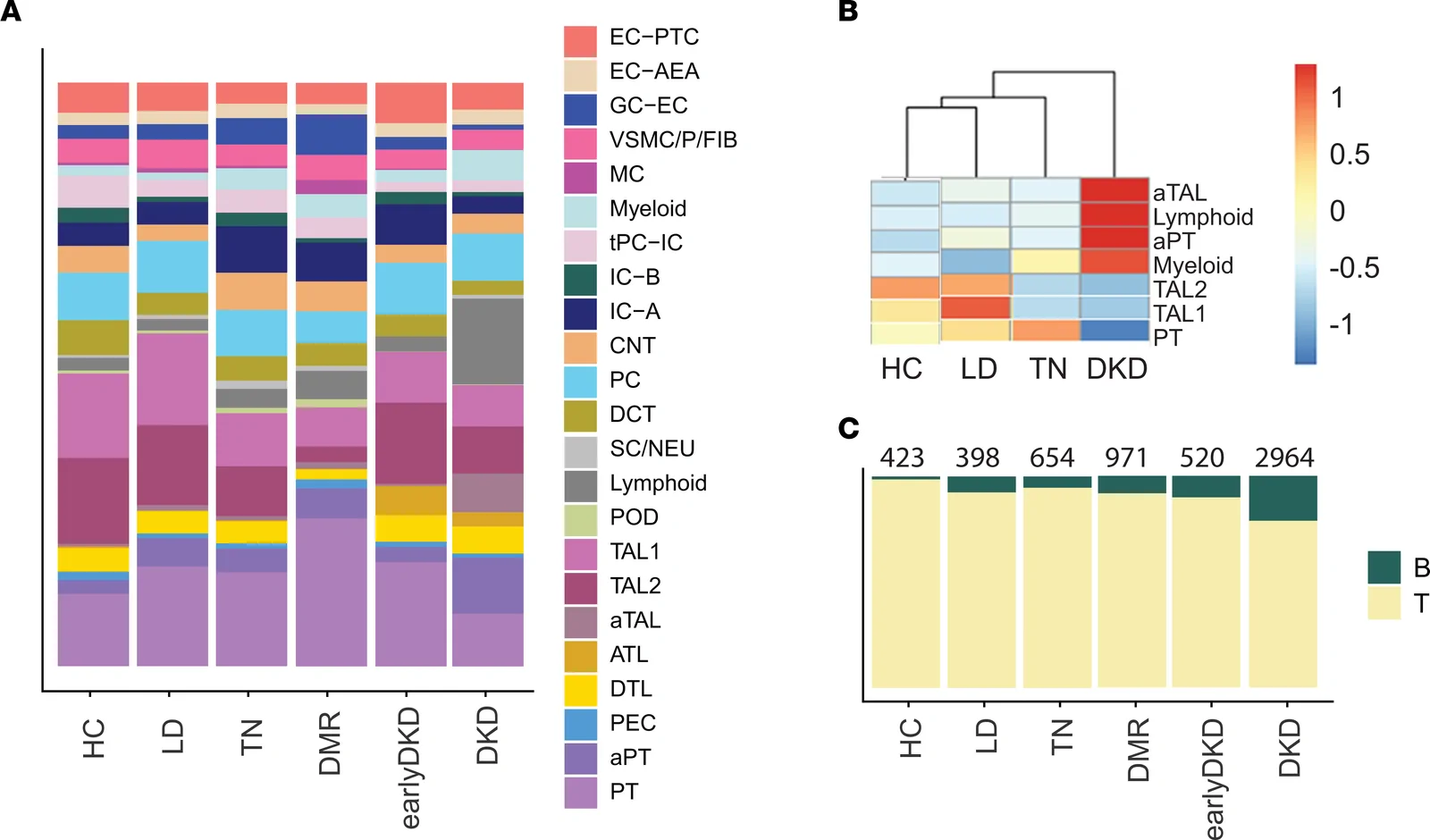
Immune cells and tubular epithelial cell states. (**A**) Proportion of cell types found in each of the 6 sample types: HC (*n* = 12), LD (*n* = 9), TN (*n* = 9), DM-R (*n* = 18), early DKD (*n* = 9), DKD (*n* = 17). (**B**) The mean proportion of PT, aPT, TAL1, TAL2, aTAL, lymphoid, and myeloid cells per sample groups studied is shown in the heatmap. The scaled expression levels show the most expression in DKD compared with reference groups. (**C**) The proportions of T and B cells within the lymphoid cell population were assessed across 6 sample groups. B cell proportion was lowest in the HC group and highest in the DKD group.

Figure 4A and Supplemental Figure 3 illustrate the proportions of cell types across sample groups. Relative to DKD, the reference groups exhibited lower proportions (adjusted *P* < 0.05) of aPT, aTAL, lymphoid, and myeloid cells, as shown in the heatmap (Figure 4B) generated from mean cell type proportions. The observed heterogeneity among aPT, aTAL, and immune cells is consistent with findings from recent single-cell studies (1, 9, 10); for example, only a very small B cell population was found in HC compared with LD and TN (Figure 4C).

#### Effect of postoperative tissue procurement (preprocessing effect).

**Figure 5:**
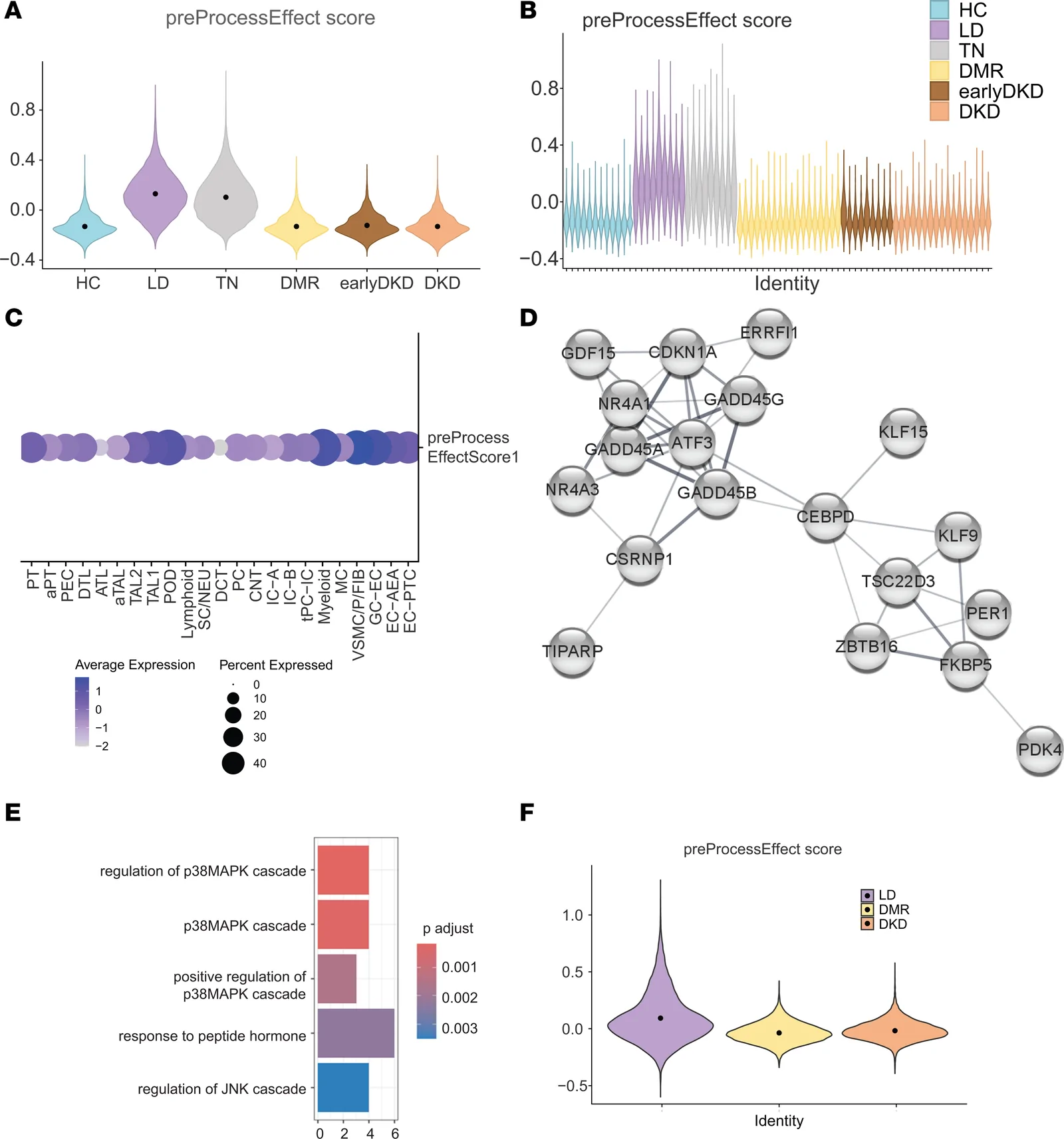
Preprocess effect. Using pseudo-bulk mRNA analysis, a gene set that was upregulated in postoperative tissue procurement method compared with percutaneous needle biopsies was identified. LD (*n* = 9) and TN (*n* = 9) samples were acquired by postoperative biopsy procedure and HC (*n* = 12), DM-R (*n* = 18), early DKD (*n* = 9), and DKD (*n* = 17) samples were by percutaneous needle biopsies. (**A**) Violin density plot showing the score calculated at cell level for the 25 genes that were upregulated in postoperative tissue biopsies. (**B**) Violin plot showing the elevated expression of the score in LD and TN samples compared with needle biopsy samples. (**C**) Dot plot showing the expression of preprocess effect score calculated based on the expression of the 25 genes in the cell types identified. (**D**) String interaction network showing direct interaction of 19/25 genes. (**E**) Top 5 enriched Gene Ontology biological processes for the gene set. (**F**) Violin plot showing preprocess effect score in Visium data from LD (*n* = 15), DM-R (*n* = 15), and DKD (*n* = 13).

Differential expression analysis between the postoperative procured sample types (LD and TN) to the percutaneous biopsy procurement (HC, all DKD samples), along with manual curation, identified a set of 25 genes (Supplemental Table 2) that exhibited significantly elevated expression in the postoperative reference samples (Figure 5, A and B). These genes were expressed in all cell types (Figure 5C). Network analysis using STRING (11) revealed direct interactions among 19 of these 25 genes (Figure 5D), with enrichment in the JNK and p38 MAPK pathways (Figure 5E). Notably, all LD and TN samples showed higher expression (adjusted *P* < 0.05) of the gene set score (computed from the 25 genes), with little intersample variability, compared with percutaneous biopsy tissues from HC and diabetic samples (Figure 5B). In the Visium integrated data, we observed high levels of preprocessing effects in the proximal cells of LD samples compared with that of DM-R and DKD samples (Figure 5F).

#### Age effect.

**Figure 6:**
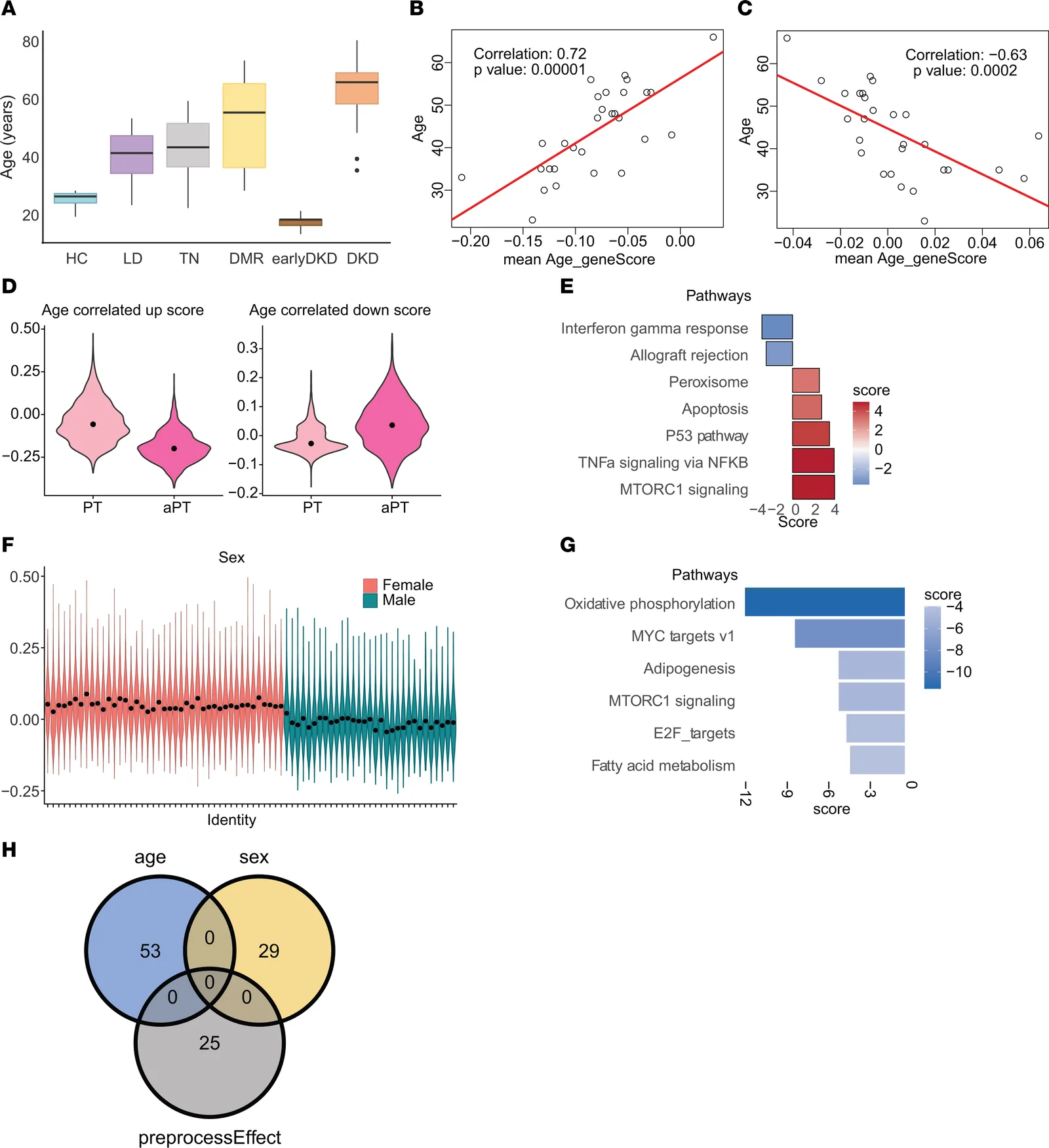
Effect of age and sex. (**A**) Bar plot showing the age distribution in each of the 6 sample groups: HC (*n* = 12), LD (*n* = 9), TN (*n* = 9), DM-R (*n* = 18), early DKD (*n* = 9), and DKD (*n* = 17). Early DKD and HC groups were much younger compared with other groups. (**B**) Plot showing the significant correlation between the scores from the positively age-associated genes and age of the living donors. (**C**) Plot showing the correlation between the scores from the negatively age-associated genes and age of the living donors. (**D**) Violin density plot showing the module scores calculated for the genes expressed in the proximal cells that are positively and negatively associated with age. The gene scores from the negatively associated genes were enriched in aPT cells, and the gene scores from the positively associated genes were higher in the PT cell state. (**E**) Top significantly enriched Hallmark MsigDB pathways for the age-associated gene set. (**F**) Violin plot illustrating higher expression scores, derived from significantly upregulated genes, in females compared with males across all 74 samples. (**G**) Top significantly enriched Hallmark MsigDB pathways for the sex-associated gene set. (**H**) The Venn diagram shows no overlap between gene sets associated with age, preprocess effect, and sex.

Figure 6A shows the age distribution per sample group. The HC and early DKD groups were considerably younger than all other sample groups. Using Pearson’s correlation analysis of pseudo-bulk read counts from both PT and aPT cells across 27 LD samples (in addition to 9 LD samples used in the integrated dataset, we used 18 LD samples from the Transplant Transcriptomic Atlas transplant study cohort, resulting in 27 samples in this analysis) (Supplemental Table 3), we identified 23 genes positively associated and 30 genes negatively associated with participant age (*P* value < 0.05) (Supplemental Table 4). Figure 6, B and C, show positive and negative correlations between age and mean sample-level Age_Gene_Scores (positive: *P* < 0.00001; negative: *P* < 0.0002). Cell-level Age_Gene_Scores were calculated from single-cell mRNA expression data for these age-correlated genes (Figure 6D). Genes positively correlated with age were enriched in healthy PT cells, and genes inversely correlated with age were more highly enriched in aPT cells. Significant (adjusted *P* < 0.05) positively and negatively enriched Hallmark pathways for the age-correlated genes are shown in Figure 6E. MTORC1 signaling, TNFA signaling via NF-κB, the p53 pathway, apoptosis, and peroxisome were significantly enriched for positive age-correlated genes. IFN-γ and allograft rejection were enriched pathways for the negative age-correlated genes.

#### Sex effect.

Differential expression analysis of samples from the 43 female and 31 male participants in the single-cell cohort, adjusted for age and preprocessing effects, identified 29 genes (adjusted *P* < 0.05) significantly enriched in females (Figure 6F and Supplemental Table 5). Gene scores calculated from the mRNA expression of these genes were consistently higher in samples from females versus males. Hallmark pathway enrichment analysis indicated that oxidative phosphorylation and MYC target pathways were significantly negatively enriched (adjusted *P* < 0.05) (Figure 6G). Figure 6H presents a Venn diagram that highlights the lack of overlap among genes correlated with preprocessing effects, age, and sex.

#### Pathway analysis.

**Figure 7:**
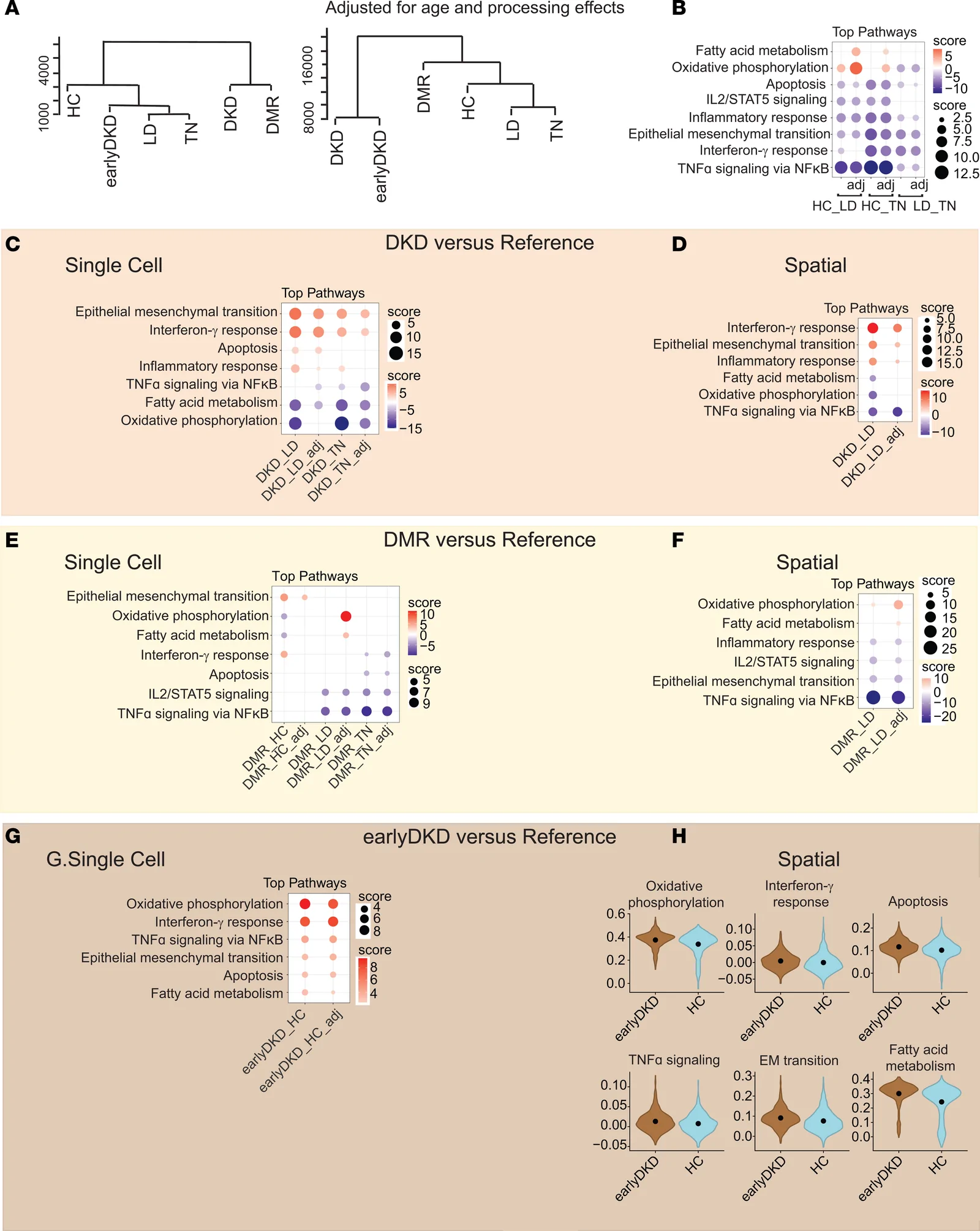
Analysis of the top pathways. (**A**) Hierarchical clustering on the right generated using aggregate read counts at the sample type level. Hierarchical clustering on the left utilized aggregate read counts with batch effect correction applied via the edgeR package. For HC, early DKD, DM-R, and DKD, age and sex were used as the covariate in batch effect correction; for TN and LD, age, process effect score, and sex served as covariates. (**B**) Plot displaying the top significant MsigDB Hallmark pathways (adjusted *P* < 0.05) associated with differentially expressed genes among the 3 reference groups. (**C**) DKD versus LD and DKD versus TN. DKD versus HC comparison was excluded due to nonoverlapping age distributions between these groups. Differential expression analysis was performed using DESeq2, both with and without adjustment for confounding variables: age, preprocess effect, and sex were included for DKD versus LD and DKD versus TN analyses. (**D**) DKD (*n* = 13) and LD (*n* = 15) in Visium data. The observed trends were consistent with those from single-cell analyses. (**E**) DM-R versus each of the 3 reference groups. Age and sex were used as the confounders for the DM-R versus HC comparison; age, sex, and preprocess effect were included for DM-R versus LD and DM-R versus TN. (**F**) DM-R (*n* = 15) versus LD (*n* = 15) in Visium data. (**G**) Early DKD versus HC. Comparisons of early DKD with other reference groups were excluded due to the absence of overlapping age distribution in these groups. Age and sex were used as the confounders for the early DKD versus HC comparison. (**H**) Violin plots depict pathway scores for selected MsigDB pathways, calculated from genes differentially expressed (*P* < 0.05) between early DKD (*n* = 4) and HC (*n* = 5) samples in Visium transcriptomic data.

Hierarchical clustering of aggregated pseudo read counts at the sample group level without adjusting for confounding factors is shown in Figure 7A. After correcting for the confounding age, sex, and preprocess effect factors, the 2 kidney disease groups, DKD and early DKD, were grouped together, and DM-R clustered with the reference groups.

#### Pathway enrichment based on differential gene expression of proximal cells among reference samples.

To characterize the molecular programs specific to different reference tissues, we performed pathway enrichment analysis on genes differentially expressed among the 3 reference groups. Differential expression analyses were conducted both with and without adjustments for age, sex, and preprocess effect. Figure 7B presents a heatmap of pathway scores for the top Hallmark pathways (adjusted *P* < 0.05) from the Molecular Signatures Database (MsigDB) across binary group comparisons.

After adjusting for confounding factors, oxidative phosphorylation and fatty acid metabolism were enriched in the HC group compared with the LD and TN groups (Figure 7B). There was no significant difference in fatty acid metabolism between the LD and TN groups, whereas there was significant reduction (adjusted *P* < 0.05) of oxidative phosphorylation in the LD group compared with the TN group (Figure 7B). Both LD and TN samples, when compared with HC, showed enrichment in apoptosis, epithelial-mesenchymal transition (EMT), TNFA signaling via NF-κB, and IL-2/STAT5 signaling pathways — even after adjustment. In the TN group compared with both LD and HC groups, pathways such as EMT, TNFA signaling via NF-κB, and IFN-γ response were enriched.

Overall, these pathway findings closely align with individual stress marker profiles, demonstrating the highest tissue differentiation and lowest stress/inflammation in HC samples, with stress and injury pathways observed in LD biopsies and, more prominently, in TN samples.

#### Pathway enrichment based on differential gene expression analysis of proximal cells between reference groups and DKD samples.

We next assessed how the choice of reference tissue influenced the enrichment of pathways shown in Figure 7B in disease versus reference comparisons.

Figure 7C illustrates significant (adjusted *P* < 0.05) Hallmark pathway enrichment for genes differentially expressed in DKD compared with reference groups (LD and TN). We excluded the DKD versus HC comparison due to the lack of age overlap between these groups. Oxidative phosphorylation and fatty acid metabolism were consistently lower in the DKD group versus the TN group, regardless of adjustment for confounding factors; however, this difference was not observed for oxidative phosphorylation between the DKD and LD groups after confounder adjustment. EMT and IFN-γ response were enriched in the DKD group relative to both reference groups, and TNFA signaling via NF-κB was reduced in the DKD group compared with the LD and TN groups, highlighting the influence of reference tissue selection on interpreting key inflammatory pathways in DKD (Figure 7C).

Figure 7D displays pathway enrichment analysis using Visium data highlighting significant (adjusted *P* < 0.05) differences between the DKD and LD groups, both before and after adjustment for confounding variables. Pathway enrichment patterns were consistent across both adjusted and unadjusted analyses in single-cell and Visium datasets. IFN-γ response remained elevated in the DKD group, corroborating findings from the single-cell data. TNFA signaling via NF-κB showed significant reduction in the DKD compared with LD group in both adjusted and unadjusted analyses (Figure 7D and Supplemental Figure 4A). Notably, differences in oxidative phosphorylation and fatty acid metabolism between the DKD and LD groups were diminished when confounding variables were considered.

Figure 7E illustrates significant (adjusted *P* < 0.05) Hallmark pathway enrichment for differentially expressed genes between the DM-R and reference groups (HC, LD, and TN). After adjusting for confounders, oxidative phosphorylation was significantly higher in DM-R than in LD. IFN-γ response enrichment in DM-R versus HC was no longer evident after adjusting for confounding factors, age, and sex. IL-2/STAT5 and TNFA signaling via NF-κB remained consistently lower in the DM-R group compared with LD and TN groups, regardless of adjustments. Results from the analysis of DM-R versus LD using Visium data further support the observations from single-cell data (Figure 7F, Supplemental Figure 3C, and Supplemental Figure 4B). Interestingly, the aPT cell state appeared to exhibit higher oxidative phosphorylation than healthy PT cells (Supplemental Figure 3C). Apoptosis, EMT, and IFN-γ response pathways were enriched in the TN group compared with other reference groups (Supplemental Figure 5) using Visium data.

Figure 7G displays the hallmark pathway enrichment for genes differentially expressed between the early DKD and HC groups. We excluded comparisons with other reference groups since there was no age overlap between early DKD and these groups. All selected pathways showed significantly higher levels (adjusted *P* < 0.05) in early DKD compared with HC. Violin density plots of the Visium data also indicated higher activity of these pathways in early DKD than in HC, though this finding should be interpreted cautiously given the small sample sizes in both groups. No-overlap between age, gene and preprocess effect were observed (Figure 7H).

## Discussion

Reference samples are essential in disease pathogenesis studies, yet obtaining pristine, healthy human kidney tissue is particularly challenging. Consequently, researchers have relied on kidney reference sources that do not closely match disease samples in terms of age or procurement protocols. To evaluate the impact of these factors on differential gene expression analyses, we compared the molecular profiles of reference samples from 3 distinct kidney tissue sources by integrating single-cell transcriptomic data from HC, LD, and TN samples. Although the samples originated from different projects, all study cohorts used identical tissue dissociation and single-cell RNA-seq protocols performed by the same KPMP Tissue Interrogation Site personnel, enabling integration without batch effects.

Kidney tissue procurement, handling, and dissociation can induce early stress responses and transcriptional changes (12). However, since all samples in this study were stored and processed using an identical protocol, observed differences are likely driven by procurement methods or donors’ physiological and clinical characteristics. Samples were obtained via percutaneous needle research biopsy (HC, early DKD, DM-R, DKD) or postoperative biopsy (LD, TN). HC samples displayed reduced expression of early stress markers compared with LD and TN samples (Figure 3A). Additionally, a gene set associated with postoperative procurement was consistently elevated in all LD and TN samples, with enriched pathways involving stress-related JNK and p38MAPK cascades that exceeded those seen in DKD biopsy tissues (Figure 5).

The HC samples had minimal or absent expression of disease markers such as *IL18*, *LCN2*, *HAVCR1*, and *VCAM1*, underscoring their pristine quality (Figure 3, B and C). Lower levels of kidney damage markers and B cell infiltration in the HC group may be attributed to both the biopsy procedure and the younger age of these donors compared with the LD and TN groups.

LD samples exhibited higher expression of *HAVCR1*, which encodes KIM-1, compared with HC and TN samples (adjusted *P* < 0.05). *HAVCR1* was significantly upregulated in the adaptive/maladaptive proximal cell state (Supplemental Figure 3A), which was prevalent in LD and TN groups (Supplemental Figure 3B). Its expression was higher in the DKD compared with LD group, remained unchanged in the DM-R versus LD group, and was significantly lower in the early DKD versus LD group. These findings suggest that age-related changes may confound the observed DKD effect in this comparison, which is consistent with age-associated elevations in KIM-1 (*HAVCR1*) reported in blood-based DKD biomarker studies (13).

TN samples consisted of noncancerous tissue from donors with high estimated glomerular filtration rate (eGFR) of more than 80, minimal pathology, and no diabetes or hypertension. IL-18, an early CKD marker and proinflammatory cytokine (14), was significantly elevated in the TN group compared with the LD and HC groups. The high enrichment of apoptosis, EMT, and IFN-γ response in the TN group compared with other reference groups (Supplemental Figure 5) that was observed in the Visium data further support the underlying stress signal present in TN samples.

We identified an age-associated gene set expressed in proximal cells, in which age-correlated genes were positively enriched for MTORC1 signaling, apoptosis, p53 signaling, and peroxisome, all known to be linked with age (15–18). Processes negatively enriched with respect to age, such as IFN-γ response and allograft rejection, have also been previously reported (19–21).

Given that confounding factors can influence transcriptomic results (22), we also reported pathway enrichment after adjusting for age, sex, and preprocess effect on gene expression profiles. This approach allowed us to correct for some of these effects intrinsic to the relatively easily obtainable TN and LD reference tissue, such as for fatty acid metabolism, and oxidative phosphorylation in the HC group compared with the LD group (Figure 7B).

The DM-R group, despite longstanding disease and risk exposure, showed minimal kidney impairment, revealing potential protective mechanisms against diabetic kidney injury. The enrichment of IFN-γ response in the DM-R group versus the HC group diminished after accounting for confounders (Figure 7D). However, oxidative phosphorylation was significantly enriched in the DM-R group compared with the LD group, most likely reflective of the higher metabolic demand in the diabetic state, as this was also observed in the early DKD kidneys (23–25).

Selection of reference tissue also affected the detection of inflammatory pathways in DKD. TNFA is well known to activate NF-κB via TNF receptor 1 (TNFR1), leading to transcription of target genes implicated in DKD (26). Both DKD versus LD and DKD versus TN showed a significant decrease in TNFA signaling via NF-κB. Notably, positive enrichment of TNFA signaling via NF-κB was only visible in the early DKD group when compared with the HC group (Supplemental Figure 4, A and B).

However, some pathways remained consistently higher in all DKD versus reference group comparisons, even after adjusting for confounders, including EMT and IFN-γ response, which are known to be robustly elevated in DKD (27, 28).

One limitation of this study is the small sample size within certain categories, resulting from the limited availability and high intrinsic value of biopsy specimens. To mitigate potential biases arising from unequal sample sizes, all groups were down-sampled to an equivalent number of cells, thereby enhancing the validity and reliability of subsequent statistical analyses.

In summary, our findings demonstrate the critical role of reference tissue sources and procurement methods for comparative gene expression analysis. In particular, the inclusion of healthy volunteer research biopsies from young adults allowed us to anchor our analyses to optimal kidney tissue. Although recruiting healthy volunteers is challenging, percutaneous needle biopsies from healthy individuals are molecularly pristine and ideal for studying biological processes across DKD stages, particularly when early or discrete disease changes are being mapped (Supplemental Table 6). However, when comparing disease biopsies from older patients, age and sex must be considered as confounding factors. LD and TN samples obtained through postsurgical biopsies are more accessible and have resulted in larger cohorts available to be studied. However, procurement stress responses affected some of the pathways altered in the disease processes of interest, thereby obscuring the disease signal. As shown in this study, confounding effects can, to some extent, be corrected by adjustment for age, sex, and preprocess effect. At a minimum, the data presented in this study will allow researchers to flag potential competing activation in both disease and reference samples in an analysis set. Finally, our study justifies the development of a framework for sufficiently diverse healthy tissue reference datasets for future analyses (29).

## Methods

### Sex as a biological variable.

Both male and female participants who were enrolled in all the cohorts were included in the analyses.

### Human data.

Key clinical and demographic characteristics of participants who contributed kidney biopsy samples in the 3 reference and 3 diabetes-related groups are summarized in Table 1. For this study, we have used data from kidney tissue collected by the Kidney Precision Medicine Project (KPMP, see https://www.kpmp.org for details), which focuses on molecular mechanisms in acute kidney injury and CKD. From the KPMP cohort, 9 LD, 17 DKD, and 18 DM-R biopsy samples were used in this study. All KPMP biopsy samples were procured at multiple repositories using a standardized protocol (30).

The TN samples comprised tumor-free kidney cortical tissue from nephrectomy specimens rapidly sourced in the operating room for molecular analyses in the PRECISE cohort, University of Michigan (31). We selected 9 TN samples from the PRECISE cohort with minimal glomerular pathology, characterized by low segmental glomerular sclerosis, global glomerular sclerosis, and imploding glomeruli. All TN samples were from participants without diabetes, with eGFR values greater than 80, and serum creatinine levels of 0.85 ± 0.15.

The 9 early DKD samples were from the Impact of Metabolic Surgery on Pancreatic, Renal and Cardiovascular Health in Youth with Type 2 Diabetes (IMPROVE-T2D) study and the Renal Hemodynamics, Energetics and Insulin Resistance in Youth Onset Type 2 Diabetes Study (Renal HEIR) and 12 control samples from research kidney biopsies performed on healthy volunteers in the Control of Renal Oxygen Consumption, Mitochondrial Dysfunction, and Insulin Resistance study (CROCODILE), as previously described (24).

HC samples and all disease biopsy samples used in the study were collected via percutaneous research needle biopsies. LD and TN samples were obtained through postoperative surgical biopsies, with LD biopsies performed on the explanted kidney before implantation into the transplant recipient.

For identifying genes associated with age using correlation analysis, we used single-cell data from tissue obtained from living donors. In addition to the 9 KPMP LD samples, we included 18 samples from the University of Michigan living donor cohort generated for the Human Kidney Transplant Transcriptomic Atlas. Immediately after procurement, tissue was transferred to a CryoStor and stored in liquid nitrogen.

### Single-cell data generation.

As a KPMP tissue interrogation site, the University of Michigan generates single-cell data from KPMP biopsy samples, which are procured at multiple repositories using a standardized protocol before being sent to Michigan. Non-KPMP datasets used in this study — including PRECISE, University of Michigan Transplant LD, and CROCODILE/ IMPROVE-T2D cohort samples — were also processed at the University of Michigan using the same protocol (https://doi.org/10.17504/protocols.io.7dthi6n) as KPMP samples. Briefly, single-cell dissociation was carried out using Liberase TL at 37°C, as described in the detailed protocol (dx.doi.org/10.17504/protocols.io.7dthi6n). The resulting single-cell suspension was immediately transferred to the University of Michigan Advanced Genomics Core for further processing. Single-cell RNA-seq was performed using 10X Genomics technology. Sequence files were aligned to the reference genome (GRCh38/hg38), and feature-barcode matrices were generated using the Cell Ranger software included in the 10X Genomics analysis suite.

### Single-cell data analysis.

The read count matrices were initially processed using SoupX (v1.5.0) to correct for ambient mRNA contamination. Data processing was performed using the Seurat R package (version 5.0). Quality control was enforced with cutoffs of greater than 500 and less than 5,000 genes per cell, and less than 50% mitochondrial reads per cell. Each sample group underwent normalization, variable gene identification, and principal component analysis (PCA) independently. Subsequently, the integration of all 6 sample types was achieved via reciprocal PCA (RPCA). Dimensionality reduction using UMAP and unsupervised clustering (resolution 0.4) were conducted on the integrated dataset. Cell clusters were annotated based on markers of major nephron segments, as well as endothelial, interstitial, and immune cell types. Further analyses were performed on a down-sampled integrated object containing 20,000 randomly selected cells from each sample type and the integrated dataset containing a total of 119,193 cells from all 6 sample types together.

### Preprocess effect analysis.

The gene set upregulated in postoperative sample types (LD and TN) compared with other groups (HC, DM-R, early DKD, and DKD) was identified via differential expression analysis using edgeR (32) on pseudo-bulk RNA read counts. The final gene set was selected after manually confirming overexpression across all samples in LD and TN groups to avoid sample-to-sample variation. The interaction network of this gene set was constructed using STRING (11) and visualized as a network in Cytoscape (33). Gene Ontology biological process enrichment (34) was performed on the preprocess effect gene set.

### Age effect.

To evaluate the impact of age within a reference group while minimizing confounding factors, we performed a correlation analysis between gene expression and donor age using 27 LD samples, as the HC and TN groups were underpowered. In addition to the 9 LD samples used in the integrated dataset, we used additional 18 LD samples from Transplant Transcriptomic Atlas transplant study cohort. Supplemental Table 3 provides age information for all samples used in the correlation analysis. We identified the top 2,500 highly variable genes in proximal cells from normalized pseudo read counts (aggregated by sample and processed with edgeR (32) and assessed their correlation with age using Pearson’s correlation test. For the positively and negatively age-correlated genes, we calculated an “Age_Gene_Score” in the integrated Seurat object using the AddModuleScore function.

### Sex effect.

To identify a set of genes that are specific to a sex, we performed a differential expression analysis on the pseudo read counts from the proximal cells from 43 female versus 31 male participants. The DESeq2 R package was used for analysis. Age and preprocess scores were used as confounding variables.

### Adjusting for age, sex, and preprocess effect in differential expression analyses.

Differential expression analyses were performed between the sample groups using the DESeq2 R package (35) on pseudo-bulk mRNA counts, generated by aggregating raw read counts from proximal cells for each sample. The analyses we conducted were both unadjusted and adjusted for age, sex, and preprocess effect as confounding factors. To account for a preprocess effect, we calculated a composite score by averaging *z* scores of 25 genes positively associated with postoperative tissue procurement. Participant age and sex at biopsy were included as covariates in the adjusted models.

### Pathway enrichment analysis.

Pathway activity enrichment was assessed using decoupleR (36) on the 2-tailed Wald-statistic values from the above-mentioned differential expression analysis of aggregated pseudo read counts from proximal cells of sample groups. Hallmark pathways from MsigDB were used as the resource for enrichment analysis. Pathways with enrichment adjusted *P* values below 0.05 were deemed significant.

Pathway enrichment analysis was initially conducted on differential expression results from the reference group comparisons. The most significantly enriched pathways (adjusted *P* < 0.05) were identified and subsequently assessed for enrichment in disease versus reference group comparisons.

### Validation of pathway activity using Visium transcriptomic data.

To assess pathway enrichment of differentially expressed genes between disease and reference groups, we analyzed transcriptomic datasets generated using 10X Genomics Visium technology. Data for LD, TN, DM-R, and DKD samples were obtained from the KPMP repository (https://atlas.kpmp.org/repository/), and HC and early DKD datasets were generated locally for an unrelated project. Human kidney tissue was prepared and imaged according to the Visium Spatial Gene Expression (10X Genomics) manufacturer’s protocol (CG000240, Visium Tissue Preparation Guide) and as previously described (1, 37).

There were 15 samples each for LD and DM-R, 13 for DKD, 5 for HC, 4 for early DKD, and 2 for TN. Because of the limited sample sizes in the HC, early DKD, and TN groups, we excluded these groups from pathway enrichment validation analyses. Instead, these groups were utilized solely for generating density plots for selected pathway gene sets.

Visium data integration was performed using Seurat. Briefly, each dataset underwent Single-Cell Transform (SCT) normalization, followed by dimensionality reduction with PCA. Integration anchors were identified using RPCA, and the datasets were merged into a single integrated object. Additional dimensionality reduction was then performed on the integrated object using both PCA and UMAP. Spot annotation was carried out with Seurat’s label transfer method, using the integrated and down-sampled single-cell dataset as the reference. For the validation of the observations from the proximal single-cell data, we focused on the Visium spots annotated as proximal (PT and aPT).

### Statistics.

To identify preprocess effect genes, we first used Seurat’s FindMarkers function to detect genes that were differentially expressed in LD and TN compared with other groups, applying an adjusted *P* value threshold of less than 0.05. Next, edgeR was performed on the pseudo-bulk read counts of these significant genes. Genes with a *P* value less than 0.05 and a log fold-change greater than 0 were then manually assessed and classified as preprocess effect genes.

Pathway activity enrichment was assessed using decoupleR on genes with significant differential expression (adjusted *P* < 0.05, using the Benjamini-Hochberg method), as identified by DESeq2 from aggregated pseudo read counts comparing proximal cells in the reference and disease groups. Pathways with enrichment adjusted *P* value less than 0.05 were considered significant.

### Study approval.

All samples used in this study were obtained with patient consent and with the approval of IRBs of participating institutions, described below. The KPMP study is reviewed and approved under a protocol by the KPMP single IRB of the University of Washington (IRB 20190213). The PRECISE cohort and the Human Kidney Transplant Transcriptomic Atlas studies were approved by the IRB of the University of Michigan. CROCODILE, Renal HEIR, and IMPROVE-T2D were approved by the Colorado Multiple IRB. Written informed consent was received from each participant and/or their parents, as appropriate for age, prior to enrollment.

### Data availability.

The count matrices of all KPMP samples used in this study are available from https://atlas.kpmp.org/repository/ The single-cell count matrices for the Human Kidney Transplant Transcriptomic Atlas are available from NCBI’s Gene Expression Omnibus (GEO GSE169285). Healthy control and early chronic disease sample read count matrices are available at GEO GSE220939 and GSE279086. The tumor nephrectomy data are at https://cellxgene.cziscience.com/collections/a98b828a-622a-483a-80e0-15703678befd Please see the Supporting Data Values file for values used to generate the figures in the main text and the supplemental figures.

## Author contributions

RM designed the study, analyzed data, interpreted results, and drafted the manuscript. MK designed the study, interpreted results, and reviewed and edited the manuscript. EAO and BG performed the single-cell experiments. PB, CLO, MB, JBH, RMF, and MTE provided transcriptomic data and interpreted the results. PLK, LS, EAO, CLO, BG, CS, FA, and CCB reviewed and edited the manuscript. YJC, LP, PL, PB, MMS, ESW, JRS, SER, SSW, and ASN provided the kidney tissue samples used in the study. All authors revised the manuscript and approved the final version.

## Conflict of interest

MK reports grants and contracts through the University of Michigan outside of this work from Chan Zuckerberg Initiative, AstraZeneca, Novo Nordisk, Eli Lilly & Co., Boehringer Ingelheim, European Union Innovative Medicine Initiative, Certa Therapeutics, Renalytix AI, Regeneron, Novo Nordisk, Sanofi, Dimerix, Travere, and Vera Therapeutics. MK has received consulting fees through the University of Michigan from Novo Nordisk, Alexion, Novartis, Roche Diagnostics, and Vera Therapeutics. In addition, MK has a patent (PCT/EP2014/073413 “Biomarkers and methods for progression prediction for chronic kidney disease”) licensed. MK also has a royalty sharing agreement for CKD drug development with AstraZeneca. PB reports serving or having served as a consultant for AstraZeneca, Bayer, Bristol Myers Squibb, Boehringer Ingelheim, Eli Lilly & Co., LG Chemistry, Merck, Sanofi, Sidera, Novo Nordisk, and Horizon Pharma. PB also serves or has served on the steering committees, advisory boards, and/or data safety committees of AstraZeneca, Bayer, Boehringer Ingelheim, Eli Lilly & Co., Sanofi, Sidera, Novo Nordisk, and XORTX Therapeutics. SER received research funding paid to her institution from Bayer. SER is a member of the steering committee for the FINE-ONE Study and attended advisory board meetings for Fresenius and Bayer. JRS has received research funding paid to his institution, for clinical trials from Vertex, Chinook, and Maze. JRS has consulted for Maze and Boehringer Ingelheim and receives royalties from Sanofi for a patent (US/11,645,753). PLK received royalties and advances from Elsevier and Mayo Clinic Press. JBH received research funding from Janssen, AstraZeneca, Gilead, and Novo Nordisk. MB has received honoraria from Wayne State University. LP has been a member of data and safety monitoring boards for 2 NIH-funded clinical trials. SSW reports research funding from Vertex, Pfizer, Johnson & Johnson, and Natera; consultancy with Wolters Kluwer, Bain, BioMarin, Goldfinch, GlaxoSmithKline, Ikena Oncology (now ImageneBio), Strataca Systems, Google, CANbridge, Novo Nordisk, Ono, PepGen, Sironax, Vertex, Mineralys Therapeutics, Motric Bio; and expert witness services for litigation related to dialysis lab testing (Davita), proton pump inhibitors (Pfizer), perfluorooctanoic acid exposure (Dechert), and a voclosporin patent (Aurinia Pharmaceuticals).

## Funding support

This work is the result of NIH funding, in whole or in part, and is subject to the NIH Public Access Policy. Through acceptance of this federal funding, the NIH has been given a right to make the work publicly available in PubMed Central.

- KPMP by the following grants from the NIH National Institute of Diabetes and Digestive and Kidney Diseases (NIDDK): U01DK133081, U01DK133091, U01DK133092, U01DK133093, U01DK133095, U01DK133097, U01DK114866, U01DK114908, U01DK133090, U01DK133113, U01DK133766, U01DK133768, U01DK114907, U01DK114920, U01DK114923, U01DK114933, U24DK114886, UH3DK114926, UH3DK114861, UH3DK114915, and UH3DK114937
- NIH funded K23 DK116720, R01 DK132399, UC2 DK114886, and P30 DK116073 (to PB).
- JDRF (2-SRA-2019-845-S-B, 3-SRA-2022-1097-M-B) (to PB).
- Boettcher Foundation (to PB).
- Intramural Research Program at NIDDK and the CDC (CKD Initiative) under interagency agreement 21FED2100157DPG (to PB).
- NIH funded Kidney Seed Network (P30 DK081943/HCA) (to MK).
- NIDDK/NCATS funded U54DK083912 (to MK).
- NIDDK funded U2C DK114886 and R21 DK126329 (to MB).
- Chan Zuckerberg Initiative (CZF2019-002447) (to MB).
- NIDDK funded K23 DK125529 (to ASN).
- George M. O’Brien Michigan Kidney Translational Resource Center funded by NIH/NIDDK grant U54DK137314 (to MK).

## Supplementary Material

Supplemental data

Supplemental tables 1-6

Supporting data values

## Figures and Tables

**Table 1:**
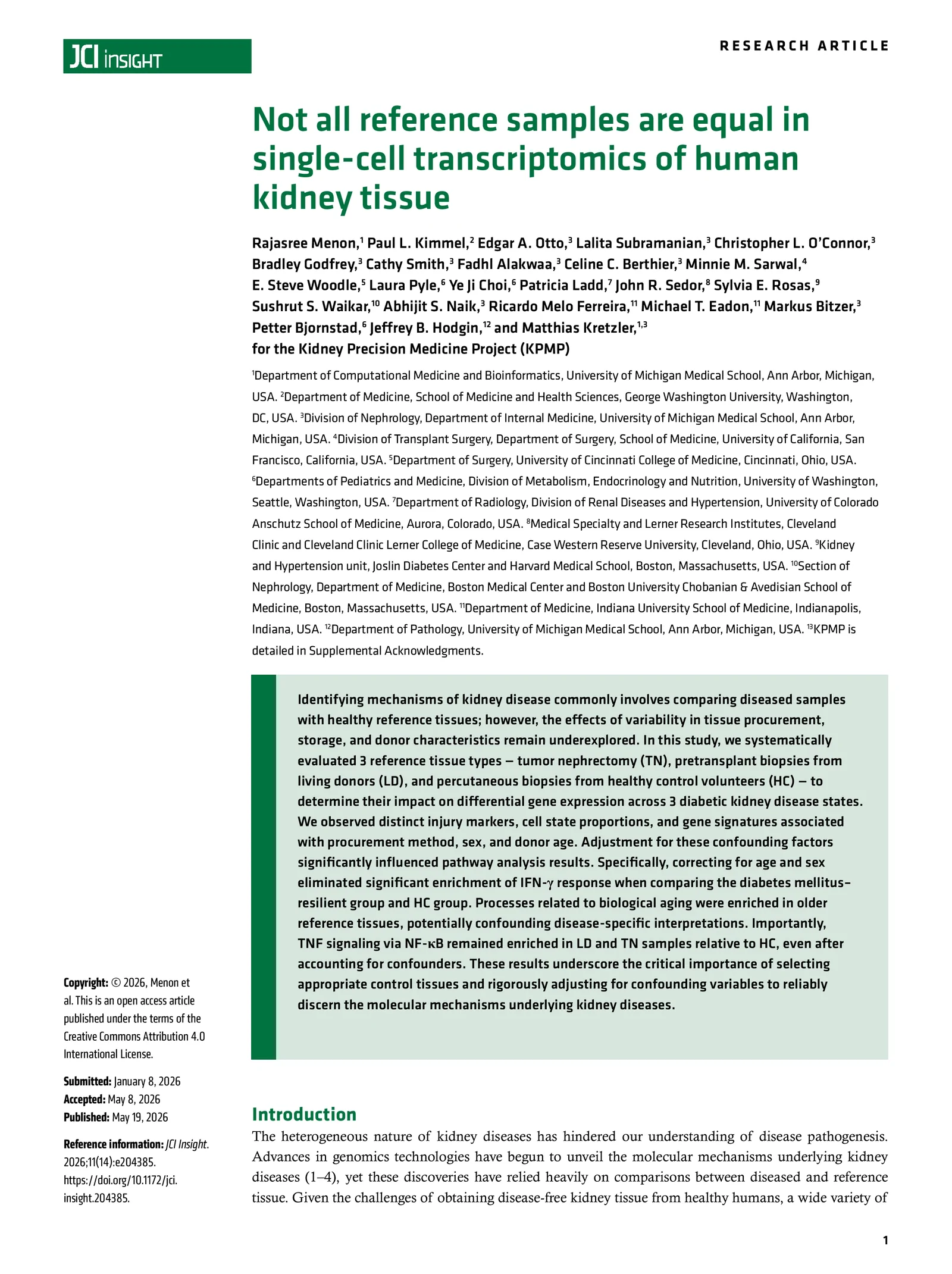
Characteristics of study participants

## Version 1 05/20/2026

In-Press Preview

## Version 2 07/22/2026

Electronic publication

## Version 3 08/26/2026

Grants from the NIH National Institute of Diabetes and Digestive and Kidney Diseases (NIDDK) included in the funding section

## References

[1] Lake BB (2023). An atlas of healthy and injured cell states and niches in the human kidney. Nature.

[2] Menon R (2022). Integrated single-cell sequencing and histopathological analyses reveal diverse injury and repair responses in a participant with acute kidney injury: a clinical-molecular-pathologic correlation. Kidney Int.

[3] Menon R (2022). Glomerular endothelial cell-podocyte stresses and crosstalk in structurally normal kidney transplants. Kidney Int.

[4] Menon R (2020). Single cell transcriptomics identifies focal segmental glomerulosclerosis remission endothelial biomarker. JCI Insight.

[5] Jain S (2023). Conundrums of choice of ‘normal’ kidney tissue for single cell studies. Curr Opin Nephrol Hypertens.

[6] Conway BR (2020). Kidney single-cell atlas reveals myeloid heterogeneity in progression and regression of kidney disease. J Am Soc Nephrol.

[7] Bahrami S, Drabløs F (2016). Gene regulation in the immediate-early response process. Adv Biol Regul.

[8] Fowler T (2011). Regulation of primary response genes. Mol Cell.

[9] Fu J (2022). The single-cell landscape of kidney immune cells reveals transcriptional heterogeneity in early diabetic kidney disease. Kidney Int.

[10] Li H (2022). Comprehensive single-cell transcriptional profiling defines shared and unique epithelial injury responses during kidney fibrosis. Cell Metab.

[11] Szklarczyk D (2019). STRING v11: protein-protein association networks with increased coverage, supporting functional discovery in genome-wide experimental datasets. Nucleic Acids Res.

[12] Denisenko E (2020). Systematic assessment of tissue dissociation and storage biases in single-cell and single-nucleus RNA-seq workflows. Genome Biol.

[13] Peng S (2022). The predicted value of kidney injury molecule-1 (KIM-1) in healthy people. Int J Gen Med.

[14] Hirooka Y, Nozaki Y (2021). Interleukin-18 in inflammatory kidney disease. Front Med (Lausanne).

[15] Di Francesco A (2018). Intermittent mTOR inhibition reverses kidney aging in old rats. J Gerontol A Biol Sci Med Sci.

[16] Xu L (2016). Inhibition of autophagy increased AGE/ROS-mediated apoptosis in mesangial cells. Cell Death Dis.

[17] Goyal K (2024). Chronic kidney disease and aging: dissecting the p53/p21 pathway as a therapeutic target. Biogerontology.

[18] Vasko R (2016). Peroxisomes and kidney injury. Antioxid Redox Signal.

[19] Ouyang Q (2000). Reduced IFN-gamma production in elderly people following in vitro stimulation with influenza vaccine and endotoxin. Mech Ageing Dev.

[20] Li G (2015). Age-associated failure to adjust type I IFN receptor signaling thresholds after T cell activation. J Immunol.

[21] Colvin MM (2017). Aging and the immune response to organ transplantation. J Clin Invest.

[22] Bruning O (2016). Confounding factors in the transcriptome analysis of an in-vivo exposure experiment. PLoS One.

[23] Bhargava P, Schnellmann RG (2017). Mitochondrial energetics in the kidney. Nat Rev Nephrol.

[24] Schaub JA (2023). SGLT2 inhibitors mitigate kidney tubular metabolic and mTORC1 perturbations in youth-onset type 2 diabetes. J Clin Invest.

[25] Burgos-Morón E (2019). Relationship between oxidative stress, ER stress, and inflammation in type 2 diabetes: the battle continues. J Clin Med.

[26] Lv J (2017). Renoprotective effect of the shen-yan-fang-shuai formula by inhibiting TNF-*α*/NF-*κ*B signaling pathway in diabetic rats. J Diabetes Res.

[27] Li Y (2019). PTEN-induced partial epithelial-mesenchymal transition drives diabetic kidney disease. J Clin Invest.

[28] Peng QY (2024). The role of immune cells in DKD: mechanisms and targeted therapies. J Inflamm Res.

[29] Kimmel Paul L, Wendler David S (2023). Biopsies from healthy volunteers to advance precision medicine. N Engl J Med.

[30] de Boer IH (2021). Rationale and design of the Kidney Precision Medicine Project. Kidney Int.

[31] Schaub JA (2022). Quantitative morphometrics reveals glomerular changes in patients with infrequent segmentally sclerosed glomeruli. J Clin Pathol.

[32] Chen Y (2025). edgeR v4: powerful differential analysis of sequencing data with expanded functionality and improved support for small counts and larger datasets. Nucleic Acids Res.

[33] Shannon P (2003). Cytoscape: a software environment for integrated models of biomolecular interaction networks. Genome Res.

[34] Yu G (2012). clusterProfiler: an R package for comparing biological themes among gene clusters. OMICS.

[35] Love MI (2014). Moderated estimation of fold change and dispersion for RNA-seq data with DESeq2. Genome Biol.

[36] Badia IMP (2022). decoupleR: ensemble of computational methods to infer biological activities from omics data. Bioinform Adv.

[37] Ferreira RM (2021). Integration of spatial and single-cell transcriptomics localizes epithelial cell-immune cross-talk in kidney injury. JCI Insight.

