# Supplementary material for: Not all reference samples are equal in single-cell transcriptomics of human kidney tissue
Source: JCI Insight. 2026 May 19;11(14):e204385. doi: 10.1172/jci.insight.204385 (PMC13461194; doi:10.1172/jci.insight.204385)
Supplement: Supplementary file 1 [file jciinsight-11-204385-s009.pdf]

Supplementary Acknowledgements

Kidney Precision Medicine Project (KPMP) members

Name	Affiliated Institution	Role
Alexander Morales	Beth Israel Deaconess Medical Center	Biopsy Operator
Mark E. Williams	Beth Israel Deaconess Medical Center	Co-Investigator
Stewart H. Lecker	Beth Israel Deaconess Medical Center	Co-Investigator,Biopsy Operator
Laurence H Beck Jr	Boston Medical Center	Co-Investigator
IM Schmidt	Boston Medical Center	Co-Investigator
Ashish Upadhyay	Boston Medical Center	Co-Investigator
Ashish Verma	Boston Medical Center	Co-Investigator
Minxin Lu	Boston Medical Center	Co-Investigator,Study staff
Yan Zhou	Boston Medical Center	Fellow
Stephanie J. Aw	Boston Medical Center	Other
Kifle Gebre	Boston Medical Center	Other
Shana Maikhor	Boston Medical Center	Other
Keyvona Moultrie	Boston Medical Center	Other
Florencia A. Rojas-Miguez	Boston Medical Center	Other
Molly C Geraghty	Boston Medical Center	Pathologist
Joel M Henderson	Boston Medical Center	Pathologist
Narasimhan, R.	Boston Medical Center	Pathologist
Sushrut S. Waikar	Boston Medical Center	Principal Investigator
Marie Florence Calixte	Boston Medical Center	Research Coordinator
Courtney Huynh	Boston Medical Center	Research Coordinator
Astrid Larson	Boston Medical Center	Research Coordinator
Ingrid F Onul	Boston Medical Center	Research Coordinator
Pranav Yadati	Boston Medical Center	Research Coordinator
Guanghao Yu, BA	Boston Medical Center	Research Coordinator
M. Todd Valerius	Brigham and Women's Hospital	Co-Investigator
Gearoid Michael McMahon	Brigham and Women's Hospital	Co-Investigator,Biopsy Operator
Mia R. Colona	Brigham and Women's Hospital	Other
Helmut Rennke	Brigham and Women's Hospital	Other
Astrid Weins	Brigham and Women's Hospital	Pathologist
Anna Greka	Broad Institute	Other
Jamie L. Marshall	Broad Institute	Other
Nir Hacohen	Broad Institute	Principal Investigator
Mark P. Aulisio	Case Western Reserve University	Co-Investigator
William S. Bush	Case Western Reserve University	Co-Investigator
Dana C. Crawford	Case Western Reserve University	Co-Investigator
Crystal A Gadegbeku	Cleveland Clinic	Co-Investigator

Jonathan J Taliercio	Cleveland Clinic	Co-Investigator
Charles O'Malley	Cleveland Clinic	Co-Investigator,Biopsy Operator
Leslie Cooperman	Cleveland Clinic	Other
Stacey Jolly	Cleveland Clinic	Other
Ellen Palmer	Cleveland Clinic	Other
Leal Herlitz	Cleveland Clinic	Pathologist
Michael Kuperman	Cleveland Clinic	Pathologist
Kassandra Spates-Harden	Cleveland Clinic	Patient Partner
John F. O'Toole	Cleveland Clinic	Principal Investigator
John R. Sedor	Cleveland Clinic	Principal Investigator
Emilio D Poggio	Cleveland Clinic	Principal Investigator,Co-Investigator
Lakeshia Bush	Cleveland Clinic	Research Coordinator
Vivian Jeffers	Cleveland Clinic	Research Coordinator
Kiasha Jones	Cleveland Clinic	Research Coordinator
Marina Markovic	Cleveland Clinic	Research Coordinator
Teresa Randle	Cleveland Clinic	Research Coordinator
Dianna Sendrey	Cleveland Clinic	Research Coordinator
Paul S. Appelbaum	Columbia University	Other
Olivia Balderes	Columbia University	Other
Pietro A. Canetta	Columbia University	Other
Karla Mehl	Columbia University	Other
Vivette . D'Agati	Columbia University	Pathologist
Jonathan Barasch	Columbia University	Principal Investigator
Andrew S. Bomback	Columbia University	Principal Investigator
Krzysztof Kiryluk	Columbia University	Principal Investigator
German varela	Columbia University	Research Coordinator
Laura Barisoni	Duke University	Co-Investigator,Pathologist
Akhil Ambekar	Duke University	Fellow
Xiang Li	Duke University	Fellow
Bangchen Wang	Duke University	Pathologist
Theodore Alexandrov	European Molecular Biology Laboratory	Co-Investigator
Jia-Yun Chen	Harvard University	Co-Investigator
Nils Gehlenborg	Harvard University	Co-Investigator
Jia-Ren Lin	Harvard University	Fellow
Yi Zhang	Harvard University	Fellow
Mark S. Keller	Harvard University	Other
Seymour Rosen	Harvard University	Pathologist
Sandro Santagata	Harvard University	Principal Investigator
Sharon B Bledsoe	Indiana University	Biopsy Operator
Ying-Hua Cheng	Indiana University	Co-Investigator

Kenneth W. Dunn	Indiana University	Co-Investigator
Michael Ferkowicz	Indiana University	Co-Investigator
K. J. Kelly	Indiana University	Co-Investigator
Ricardo Melo Ferreira	Indiana University	Co-Investigator
Marcelino Rivera	Indiana University	Co-Investigator
Timothy A. Sutton	Indiana University	Co-Investigator
Debora Gisch	Indiana University	Co-Investigator,Fellow
Azuma Nanamatsu	Indiana University	Fellow
Mohammad A. Sohail	Indiana University	Fellow
Mahla Asghari	Indiana University	Other
Andreas Bueckle	Indiana University	Other
Curtis Warfield	Indiana University	Patient Partner
Tarek M. El-Achkar	Indiana University	Principal Investigator
Pierre C. Dagher	Indiana University	Principal Investigator
Michael T Eadon	Indiana University	Principal Investigator
Danielle Janosevic	Indiana University	Principal Investigator
James C. Williams, Jr.	Indiana University	Principal Investigator
Abraham Verdoes	Indiana University	Project Manager
Devin M. Wright	Indiana University	Project Manager
Stephanie Wofford	Indiana University	Research Coordinator
Daria Barwinska	Indiana University	Study staff
William S. Bowen	Indiana University	Study staff
Angela R. Sabo	Indiana University	Study staff
Jennifer Stashevsky	Indiana University	Study staff
Katy Börner	Indiana University Bloomington	Other
Ellen M. Quardokus	Indiana University Bloomington	Other
Jini Ashok Bhanushali	Indiana University Bloomington	Project Manager
Bruce W. Herr II	Indiana University Bloomington	Project Manager,Other
Elizabeth G. Record	Indiana University Bloomington	Research Coordinator
Derek M. Fine	Johns Hopkins University	Biopsy Operator
C. John Sperati	Johns Hopkins University	Biopsy Operator
Steven Menez	Johns Hopkins University	Co-Investigator
Mohamed G. Atta	Johns Hopkins University	Co-Investigator,Biopsy Operator
Jose M Monroy-Trujillo	Johns Hopkins University	Co-Investigator,Biopsy Operator
Yumeng Wen	Johns Hopkins University	Co-Investigator,Fellow
Lauren Bernard	Johns Hopkins University	Other
Noralinda B. Viloria	Johns Hopkins University	Other
Alan Xu	Johns Hopkins University	Other
Avi Z. Rosenberg	Johns Hopkins University	Pathologist
Chirag R. Parikh	Johns Hopkins University	Principal Investigator
Celia P. Corona-Villalobos	Johns Hopkins University	Project Manager
Mitali Barik	Johns Hopkins University	Research Coordinator

Maria Chilo Bejarano	Johns Hopkins University	Research Coordinator
Jeanine Hernandez	Johns Hopkins University	Research Coordinator
Sonya Shah	Johns Hopkins University	Research Coordinator
Ashley R. Wang	Johns Hopkins University	Research Coordinator
Paolo S. Silva	Joslin Diabetes Center	Co-Investigator
Jennifer K. Sun	Joslin Diabetes Center	Co-Investigator
Isabel Donohoe	Joslin Diabetes Center	Other
Camille Johansen	Joslin Diabetes Center	Other
N/A	Joslin Diabetes Center	Other
Rosas, Sylvia E.	Joslin Diabetes Center	Principal Investigator
Sophia A. Angus	Joslin Diabetes Center	Research Coordinator
Sarah W Chen	Joslin Diabetes Center	Research Coordinator
Asari Henshaw	Joslin Diabetes Center	Research Coordinator
Neil Roy	Joslin Diabetes Center	Research Coordinator
Imane H. Samari	Joslin Diabetes Center	Research Coordinator
Gabriel Zeinoun	Joslin Diabetes Center	Research Coordinator
Mallory Mandel	Joslin Diabetes Center	Study staff
Jenny Molina-Guzman	Joslin Diabetes Center	Study staff
Anna Kate Stawicki	Joslin Diabetes Center	Study staff
Melissa D. Rubinsky	Joslin Diabetes Center	Study staff,Other
Julia A. Welch	Joslin Diabetes Center	Study staff,Other
Lili Chan	Mount Sinai	Biopsy Operator
Daniel Stalbow	Mount Sinai	Biopsy Operator
Evren U Azeloglu	Mount Sinai	Co-Investigator
Jens Hansen	Mount Sinai	Co-Investigator
John Cijiang He	Mount Sinai	Co-Investigator
Carol R. Horowitz	Mount Sinai	Co-Investigator
Ravi Iyengar	Mount Sinai	Co-Investigator
Joji Tokita	Mount Sinai	Co-Investigator,Biopsy Operator
Patricia Kovatch	Mount Sinai	Co-Investigator,Other
Marina de Cos	Mount Sinai	Fellow
Jonathan Haydak	Mount Sinai	Fellow,Patient Partner
Lili Gai	Mount Sinai	Other
Patricia Kovatch	Mount Sinai	Other
Timothy D. Quinn	Mount Sinai	Other
Ronald E. Gordon	Mount Sinai	Pathologist
Ritu Gupta	Mount Sinai	Pathologist
Isaac E Stillman	Mount Sinai	Pathologist
Stephen C Ward	Mount Sinai	Pathologist
Kirk N Campbell	Mount Sinai	Principal Investigator
Steven G. Coca	Mount Sinai	Principal Investigator
Jonathan Himmelfarb	Mount Sinai	Principal Investigator
Kristin Meliambro	Mount Sinai	Principal Investigator

Girish N Nadkarni	Mount Sinai	Principal Investigator
Mark L Green	Mount Sinai	Project Manager
Brandon G Larson	Mount Sinai	Project Manager
Lorraine Evo-Ortega	Mount Sinai	Research Coordinator
Gina Koch	Mount Sinai	Research Coordinator
Marissa Patel	Mount Sinai	Research Coordinator
Sean Lefferts	Mount Sinai	Research Coordinator,Project Manager
Tejas Rao	Mount Sinai	Study staff
Samuel Mon-Wei Yu	Mount Sinai	Study staff
Pottumarthi V Prasad	Northwestern University	Co-Investigator
Samir V Parikh	Ohio State University	Co-Investigator
Brad H. Rovin	Ohio State University	Principal Investigator
Gek Cher Chan	Other	Co-Investigator
Yijiang Chen	Other	Co-Investigator
Joana P. Gonçalves	Other	Co-Investigator
Andrew Janowczyk	Other	Co-Investigator
Blue B. Lake	Other	Co-Investigator
Roy Lardenoije	Other	Co-Investigator
Rosamond Rhodes	Other	Co-Investigator
Milda R. Saunders	Other	Co-Investigator
Jovan Tanevski	Other	Co-Investigator
Seth Winfree	Other	Co-Investigator
Robin Fallegger	Other	Fellow
Steve Bogen	Other	Other
Charlotte Boys	Other	Other
Brandon Ginley	Other	Other
Leah Guthrie	Other	Other
Leonie Küchenhoff	Other	Other
Brendon Lutnick	Other	Other
Joseph Ardayfio	Other	Patient Partner,Other
Jack Bebiak	Other	Patient Partner,Other
Taneisha Campbell	Other	Patient Partner,Other
Robert Koewler	Other	Patient Partner,Other
Roy Pinkeney	Other	Patient Partner,Other
John Saul	Other	Patient Partner,Other
Dongwon Lee	Other	Principal Investigator
Anant Madabhushi	Other	Principal Investigator
Ari Pollack	Other	Principal Investigator
Julio Saez-Rodriguez	Other	Principal Investigator
Raf Van de Plas	Other	Principal Investigator
Ashley R Burg	Other	Research Coordinator,Study staff
Lukasz G. Migas	Other	Study staff

Ljiljana Paša-Tolić	Pacific Northwest National Laboratory	Co-Investigator
Dusan Velickovic	Pacific Northwest National Laboratory	Co-Investigator
Jessica Lukowski	Pacific Northwest National Laboratory	Other
Christopher R Anderton	Pacific Northwest National Laboratory	Principal Investigator
Kisurb Choe	Pacific Northwest National Laboratory	Study staff
Brittney L. Gorman	Pacific Northwest National Laboratory	Study staff
George (Holt) Oliver	Parkland Health and Hospital System	Other
Rachel S. G. Sealfon	Princeton University	Co-Investigator
Xi Chen	Princeton University	Other
Weiguang Mao	Princeton University	Other
Ksenia Sokolova	Princeton University	Other
Aaron Wong	Princeton University	Other
Olga G Troyanskaya	Princeton University	Principal Investigator
David H. Beyda, MD	University of Arizona	Co-Investigator
thajudeen b	University of Arizona	Co-Investigator
Rebecca Tsosie	University of Arizona	Co-Investigator
Gregory Woodhead	University of Arizona	Co-Investigator,Biopsy Operator
Erika R Bracamonte	University of Arizona	Pathologist
Raymond Scott	University of Arizona	Patient Partner
Frank C. Brosius	University of Arizona	Principal Investigator
Baltazar Campos	University of Arizona	Research Coordinator
Austin Derma	University of Arizona	Research Coordinator
Daniel Damian Duran	University of Arizona	Research Coordinator
Griselda Gamez	University of Arizona	Research Coordinator
Nicole Marquez	University of Arizona	Research Coordinator
Katherine Mendoza	University of Arizona	Research Coordinator
Ana Celina Sanora	University of Arizona	Research Coordinator
Kun Zhang	University of California San Diego	Co-Investigator
Tara K Sigdel	University of California San Francisco	Co-Investigator
Kavya Anjani	University of California San Francisco	Other
Tariq Mukatash	University of California San Francisco	Other
Zoltan G. Laszik	University of California San Francisco	Principal Investigator
Minnie M Sarwal	University of California San Francisco	Principal Investigator
James G. Cimino	University of California San Francisco	Research Coordinator
Dane Munar	University of California San Francisco	Research Coordinator
Laura Pyle	University of Colorado	Co-Investigator
Julia Wrobel	University of Colorado	Co-Investigator
Carissa Vinovskis	University of Colorado	Other
Petter Bjornstad	University of Colorado	Principal Investigator
Hsieh EWY	University of Colorado	Principal Investigator
Joshua M. Thurman	University of Colorado	Principal Investigator
Pinaki Sarder	University of Florida	Co-Investigator
Manoj Kumar Galla	University of Florida	Other

Harshit Lohaan	University of Florida	Other
Sayat Mimar	University of Florida	Other
Samuel Border	University of Florida	Study staff
Nicholas Lucarelli	University of Florida	Study staff
Ahmed Naglah	University of Florida	Study staff
Anindya S. Paul	University of Florida	Study staff
Michael Tanious	University of Illinois, Chicago	Biopsy Operator
Tanika N. Kelly	University of Illinois, Chicago	Co-Investigator
Bui, JT or Bui, James T	University of Illinois, Chicago	Co-Investigator,Biopsy Operator
Ron C. Gaba	University of Illinois, Chicago	Co-Investigator,Biopsy Operator
Aaron Scroggins	University of Illinois, Chicago	Other
Suman Setty	University of Illinois, Chicago	Pathologist
Monica L. Fox	University of Illinois, Chicago	Patient Partner
James P. Lash	University of Illinois, Chicago	Principal Investigator
Ana C. Ricardo	University of Illinois, Chicago	Principal Investigator
Anand Srivastava	University of Illinois, Chicago	Principal Investigator,Co-Investigator,Other
Joed Ancheta	University of Illinois, Chicago	Research Coordinator
Eunice Carmona-Powell	University of Illinois, Chicago	Research Coordinator
Natalie Meza	University of Illinois, Chicago	Research Coordinator
Arabela Quiroga	University of Illinois, Chicago	Research Coordinator
Amada Renteria	University of Illinois, Chicago	Research Coordinator
Kim Silva	University of Illinois, Chicago	Research Coordinator,Project Manager
Devona Redmond	University of Illinois, Chicago	Study staff,Other
Fadhl Alakwaa	University of Michigan	Co-Investigator
Ulysses G. J. Balis	University of Michigan	Co-Investigator
Markus Bitzer	University of Michigan	Co-Investigator
Yongqun He	University of Michigan	Co-Investigator
Wenjun Ju	University of Michigan	Co-Investigator
Laura H. Mariani	University of Michigan	Co-Investigator
Rajasree Menon	University of Michigan	Co-Investigator
Abhijit S. Naik	University of Michigan	Co-Investigator
Edgar A. Otto	University of Michigan	Co-Investigator
Jennifer A. Schaub	University of Michigan	Co-Investigator
Dawit Demeke	University of Michigan	Fellow
Francesca Annese	University of Michigan	Other
Heather K. Ascani	University of Michigan	Other
Victoria M. Blanc	University of Michigan	Other
Nathan Creger	University of Michigan	Other
Rachel Dull	University of Michigan	Other
Renee Frey	University of Michigan	Other
Josh Hartley	University of Michigan	Other

Chrysta C Lienczewski	University of Michigan	Other
Becky Steck	University of Michigan	Other
Haneen Tout	University of Michigan	Other
Zach Wright	University of Michigan	Other
Matthias Kretzler	University of Michigan	Principal Investigator
Jeffrey B. Hodgin	University of Michigan	Principal Investigator,Co-Investigator,Pathologist
Lalita Subramanian	University of Michigan	Project Manager
Nikole Bonevich	University of Michigan	Project Manager,Other
Ninive Conser	University of Michigan	Study staff
Sean Eddy	University of Michigan	Study staff
John Hartman	University of Michigan	Study staff
Phillip J. McCown	University of Michigan	Study staff
Viji Nair	University of Michigan	Study staff
Rebecca Reamy	University of Michigan	Study staff
Michael P. Rose	University of Michigan	Study staff
Cathy Smith	University of Michigan	Study staff
Donna D'Souza	University of Minnesota	Biopsy Operator
Siobhan M. Flanagan	University of Minnesota	Biopsy Operator
Jerica M. Berge	University of Minnesota	Co-Investigator
Drawz PE	University of Minnesota	Co-Investigator
Tasma Harindhanavudhi	University of Minnesota	Co-Investigator
Sisi Ma	University of Minnesota	Co-Investigator
Elizabeth A. Rogers	University of Minnesota	Co-Investigator
Michael S. Rosenberg	University of Minnesota	Co-Investigator
Sami Safadi	University of Minnesota	Co-Investigator
Susan M. Wolf	University of Minnesota	Co-Investigator
Christopher J. Jones	University of Minnesota	Co-Investigator,Biopsy Operator
Sandeep Sharma	University of Minnesota	Co-Investigator,Biopsy Operator
Oyedele A. Adeyi	University of Minnesota	Pathologist
Yanli Ding	University of Minnesota	Pathologist
Ann Gentry	University of Minnesota	Patient Partner
Susan Klett	University of Minnesota	Patient Partner
M. Luiza Caramori	University of Minnesota	Principal Investigator
Patrick H. Nachman	University of Minnesota	Principal Investigator
Alyson Coleman	University of Minnesota	Project Manager
Dori Henderson	University of Minnesota	Project Manager
Cathy A Bagne	University of Minnesota	Research Coordinator
Rachel R. Kaspari	University of Minnesota	Research Coordinator
Oluwatosin Oluwole	University of Minnesota	Research Coordinator
Via Rao	University of Minnesota	Research Coordinator
Nicolas J Rauwolf	University of Minnesota	Research Coordinator
Michelle L. Snyder	University of Minnesota	Research Coordinator

Zoe Wright	University of Minnesota	Research Coordinator
Alison Bunio Alvear	University of Minnesota	Study staff
Peter R. Bream, Jr.	University of North Carolina	Biopsy Operator
Nicole Keefe	University of North Carolina	Biopsy Operator
Priya Mody	University of North Carolina	Biopsy Operator
Saad Mohammed Shariff	University of North Carolina	Biopsy Operator
Alexander Villalobos	University of North Carolina	Biopsy Operator
Prabir Roy-Chaudhury	University of North Carolina	Co-Investigator
Evan M. Zeitler	University of North Carolina	Co-Investigator
Vanessa Moreno	University of North Carolina	Co-Investigator,Pathologist
Samuel Haddad	University of North Carolina	Other
J Charles Jennette	University of North Carolina	Pathologist
Jennifer L. Jones	University of North Carolina	Patient Partner
Amy K. Mottl	University of North Carolina	Principal Investigator
Tashas Cameron-Wheeler	University of North Carolina	Research Coordinator
Mary M. Collie	University of North Carolina	Research Coordinator
Anne Froment	University of North Carolina	Research Coordinator
Dhatri Kakarla	University of North Carolina	Research Coordinator
Sara S. Kelley	University of North Carolina	Research Coordinator
Sora Lee	University of North Carolina	Research Coordinator
Fernanda Ochoa Toro	University of North Carolina	Research Coordinator
Sandhya Sundar Rajan	University of North Carolina	Research Coordinator,Study staff
Matthew Gilliam	University of Pittsburgh	Other
Daniel E. Hall	University of Pittsburgh	Other
John A. Kellum	University of Pittsburgh	Other
Roderick Tan	University of Pittsburgh	Other
James Winters	University of Pittsburgh	Other
Parmjeet Randhawa	University of Pittsburgh	Pathologist
Raghavan Murugan	University of Pittsburgh	Principal Investigator
Paul M. Palevsky	University of Pittsburgh	Principal Investigator
Matthew R. Rosengart	University of Pittsburgh	Principal Investigator
Michele M Elder	University of Pittsburgh	Project Manager
Adam Burgess	University of Pittsburgh	Research Coordinator
Tina Vita	University of Pittsburgh	Research Coordinator
Soumya Maity	University of Texas Health Science Center at San Antonio	Co-Investigator
Guanshi Zhang	University of Texas Health Science Center at San Antonio	Co-Investigator
manjeri venkatachalam	University of Texas Health Science Center at San Antonio	Co-Investigator,Pathologist
Bhupendra Kumar Gurung	University of Texas Health Science Center at San Antonio	Other

Annapurna Pamreddy	University of Texas Health Science Center at San Antonio	Other
Hongping Ye	University of Texas Health Science Center at San Antonio	Other
Shiqi Zhang	University of Texas Health Science Center at San Antonio	Other
Kumar Sharma	University of Texas Health Science Center at San Antonio	Principal Investigator
NAGARJUNACHARY RAGI	University of Texas Health Science Center at San Antonio	Study staff
Samuel Rice	University of Texas Southwestern	Biopsy Operator
S. Susan Hedayati	University of Texas Southwestern	Co-Investigator
Meredith C McAdams	University of Texas Southwestern	Co-Investigator
R. Tyler Miller	University of Texas Southwestern	Co-Investigator
Jiten Patel	University of Texas Southwestern	Co-Investigator
Choudhary Moaz	University of Texas Southwestern	Co-Investigator,Biopsy Operator
Sanjeeva P. Kalva	University of Texas Southwestern	Co-Investigator,Biopsy Operator
Allen R Hendricks	University of Texas Southwestern	Co-Investigator,Pathologist
Asra Kermani MD	University of Texas Southwestern	Other
Simon C. Lee	University of Texas Southwestern	Other
Harold Park	University of Texas Southwestern	Other
Anil Pillai	University of Texas Southwestern	Other
Natasha Wen	University of Texas Southwestern	Other
Qi Cai	University of Texas Southwestern	Pathologist
Jose R. Torrealba	University of Texas Southwestern	Pathologist
Catherine Campbell	University of Texas Southwestern	Patient Partner
Robert D Toto	University of Texas Southwestern	Principal Investigator
Miguel A. Vazquez	University of Texas Southwestern	Principal Investigator
Shihong Ma	University of Texas Southwestern	Research Coordinator
Nancy Wang	University of Texas Southwestern	Research Coordinator
Boris S. Patlis	University of Texas Southwestern	Research Coordinator,Other
Andrew N Hoofnagle	University of Washington	Co-Investigator
Robyn L. McClelland	University of Washington	Co-Investigator
Kasra A Rezaei	University of Washington	Co-Investigator
Jaime Snyder	University of Washington	Co-Investigator
Katherine R. Tuttle	University of Washington	Co-Investigator
Bessie A. Young	University of Washington	Co-Investigator
Christine P Limonte	University of Washington	Co-Investigator,Fellow
Ruikang Wang	University of Washington	Co-Investigator,Other
Stephanie M. Grewenow	University of Washington	Other
Cienn N. Joyeux	University of Washington	Other
Yunbi Nam	University of Washington	Other
Christopher Park	University of Washington	Other

Adam Wilcox	University of Washington	Other
Kayleen Williams	University of Washington	Other
CE Alpers	University of Washington	Pathologist
Kelly D. Smith	University of Washington	Pathologist
Keith D. Brown	University of Washington	Patient Partner
Lynda Hayashi	University of Washington	Patient Partner
Nichole M. Jefferson	University of Washington	Patient Partner
Richard A. Knight	University of Washington	Patient Partner
Glenda V. Roberts	University of Washington	Patient Partner
Christy Stutzke	University of Washington	Patient Partner
Ian H. de Boer	University of Washington	Principal Investigator,Co-Investigator
Brooke Berry	University of Washington	Project Manager
Kristina N Blank	University of Washington	Project Manager
Ashveena L Dighe	University of Washington	Project Manager
Alexa Plisiewicz	University of Washington	Project Manager
Natalya Sarkisova	University of Washington	Project Manager
Ashley C Berglund	University of Washington	Study staff
Jonas M Carson	University of Washington	Study staff
Matthew Dekker	University of Washington	Study staff
Frederick Dowd	University of Washington	Study staff
Jimmy Phuong	University of Washington	Study staff
Artit Wangperawong	University of Washington	Study staff
de Caestecker M.P.	Vanderbilt University	Co-Investigator
Agnes B. Fogo	Vanderbilt University	Co-Investigator,Pathologist
Katerina V. Djambazova	Vanderbilt University	Fellow
Audra M. Judd	Vanderbilt University	Other
Yarieli Cuevas-Rios	Vanderbilt University	Patient Partner
Yuankai Huo	Vanderbilt University	Principal Investigator
Jeffrey M. Spraggins	Vanderbilt University	Principal Investigator
Melissa A Farrow	Vanderbilt University	Project Manager
Jamie L Allen	Vanderbilt University	Study staff
Madeline E. Colley	Vanderbilt University	Study staff
Ruining Deng	Vanderbilt University	Study staff
Martin Dufresne	Vanderbilt University	Study staff
Angela R.S. Kruse	Vanderbilt University	Study staff
Jeannine Basta	Washington University St. Louis	Co-Investigator
Anitha Vijayan	Washington University St. Louis	Co-Investigator
Reetika Ghag	Washington University St. Louis	Other
Amanda Knoten	Washington University St. Louis	Other
Asmita L	Washington University St. Louis	Other
Stephanie Reinert	Washington University St. Louis	Other
Joseph P. Gaut	Washington University St. Louis	Pathologist

Michael Rauchman	Washington University St. Louis	Principal Investigator
Sanjay Jain	Washington University St. Louis	Principal Investigator,Other
Madhurima Kaushal	Washington University St. Louis	Project Manager,Study staff
Amy McMurray	Washington University St. Louis	Research Coordinator
Kristine Conlon	Washington University St. Louis	Research Coordinator,Patient Partner
Brittany C Minor	Washington University St. Louis	Study staff
Gerald Nwanne	Washington University St. Louis	Study staff
Bo Zhang	Washington University St. Louis	Study staff
Jeffrey M Turner	Yale University	Biopsy Operator
Moledina DG	Yale University	Co-Investigator
Tanima Arora	Yale University	Other
Gilbert W. Moeckel	Yale University	Pathologist
Vijayakumar R Kakade	Yale University	Principal Investigator
Lloyd G Cantley	Yale University	Principal Investigator,Co-Investigator
F. Perry Wilson	Yale University	Principal Investigator,Co-Investigator
Ugochukwu Ugwuowo	Yale University	Research Coordinator
Angela M. Victoria-Castro	Yale University	Research Coordinator
Melissa M. Shaw	Yale University	Research Coordinator,Study staff
Tifanny Budiman	Yale University	Study staff

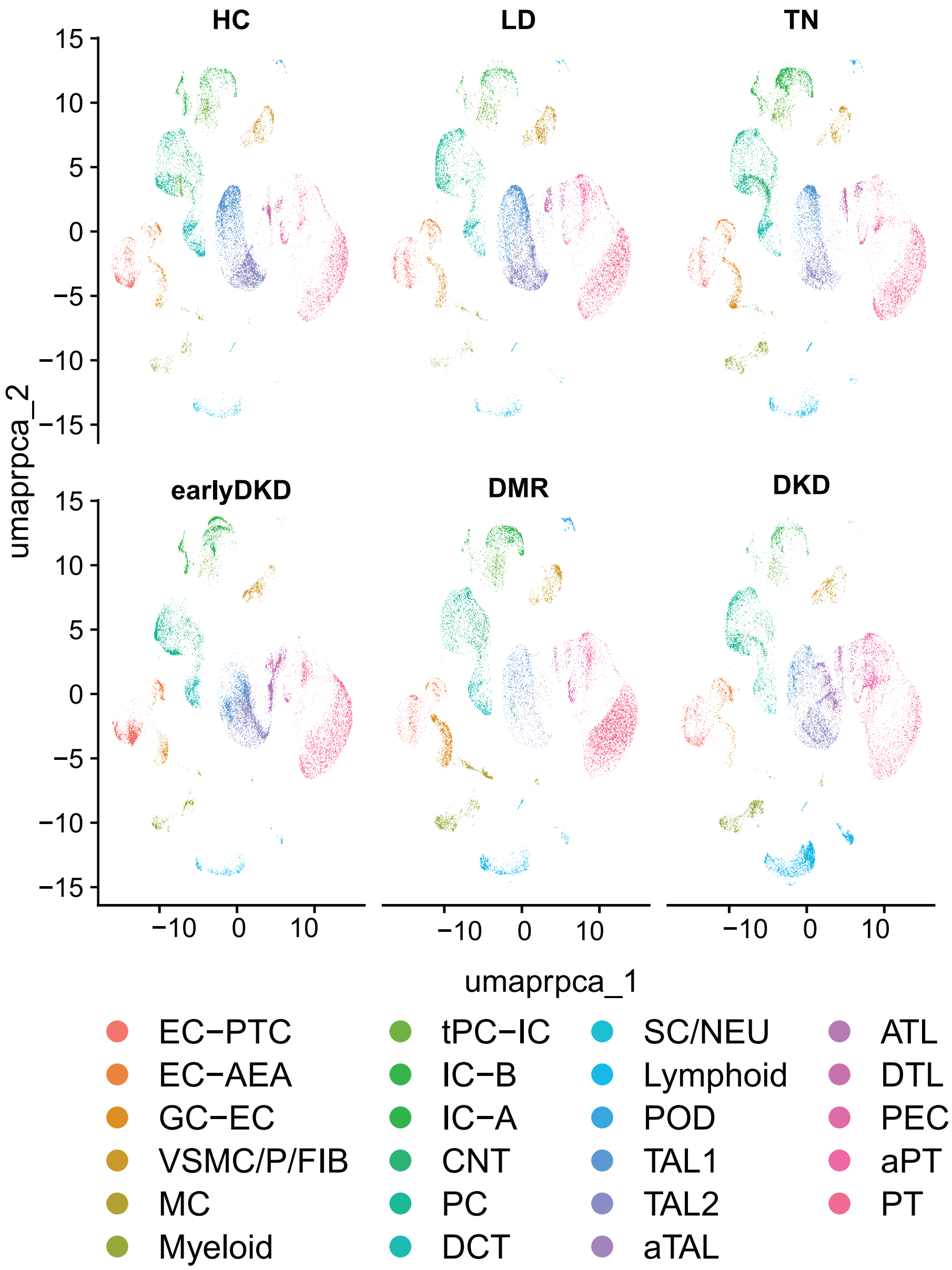

Supplementary Figure S1. Split UMAP of the 6 sample groups studied
Abbreviations: HC- healthy controls; LD- living kidney donor; TN- tumor nephrectomy; DMR - diabetes mellitus resistor; DKD - diabetic kidney disease;
Cell types: podocyte, POD; parietal epithelial cell, PEC; proximal tubule, PT; adaptive/maladaptive, aPT; descending thin loop of Henle, DTL; ascending thin loop of Henle, ATL; thick ascending loop of Henle, TAL; adaptive/maladaptive thick ascending loop of Henle, aTAL; distal convoluted tubule, DCT; connecting tubule, CNT; principal cell, PC; intercalated type A, IC-A; Intercalated type B, IC-B; transient between PC and IC, tPC-IC; endothelial cell EC; efferent and afferent arteriolar endothelial cells EC-AEA; glomerular endothelial cell, EC-GC; fibroblast, FIB; vascular smooth muscle cell, VSMC; pericyte, P; mesangial cell, MC; myeloid cell, MYL; B cell, B; T cell, T; natural killer T cell, NK T/C.

Supplementary Figure S2

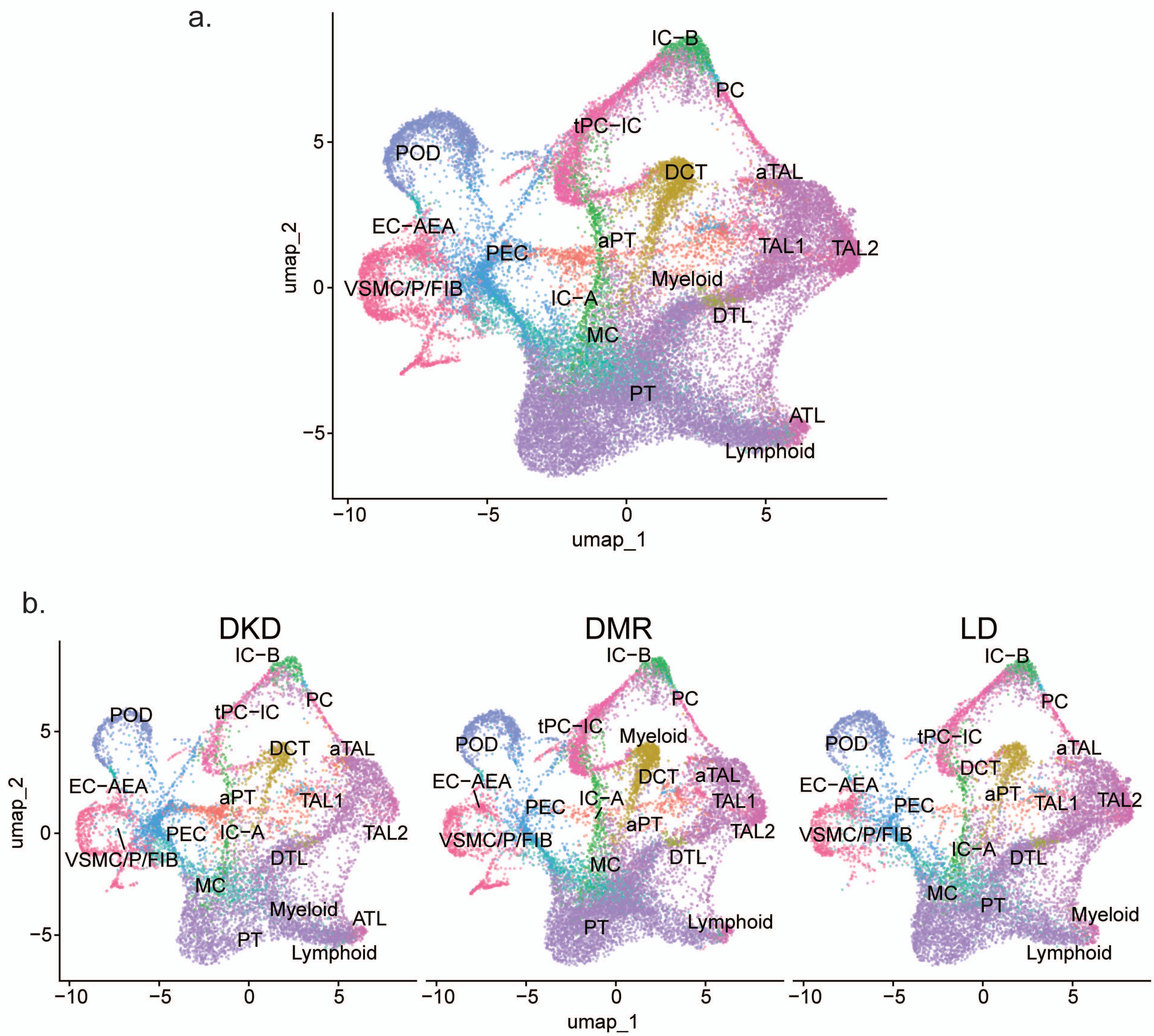

Supplementary Figure S2. UMAPs of Visium data. a. Combined and **b.**split UMAPs of the integrated Visium dataset containing LD, DMR-R and DKD samples. These datasets were downloaded from KPMP repository site., 15 samples per LD, and DM-R, and 13 samples in DKD. 38,942 spots in total from the three sample groups.

Abbreviations: Living donor LD, Diabetes mellitus resilient DM-R, Diabetic kidney disease DKD

Cell types: podocyte, POD; parietal epithelial cell, PEC; proximal tubule, PT; adaptive/maladaptive, aPT; descending thin loop of Henle, DTL; ascending thin loop of Henle, ATL; thick ascending loop of Henle, TAL; adaptive/maladaptive thick ascending loop of Henle, aTAL; distal convoluted tubule, DCT; connecting tubule, CNT; principal cell, PC; intercalated type A, IC-A; Intercalated type B, IC-B; transient between PC and IC, tPC-IC; endothelial cell EC; efferent and afferent arteriolar endothelial cells EC-AEA; glomerular endothelial cell, EC-GC; fibroblast, FIB; vascular smooth muscle cell, VSMC; pericyte, P; mesangial cell, MC; myeloid cell, MYL; B cell, B; T cell, T; natural killer T cell, NK T/C.

Supplementary Figure S3

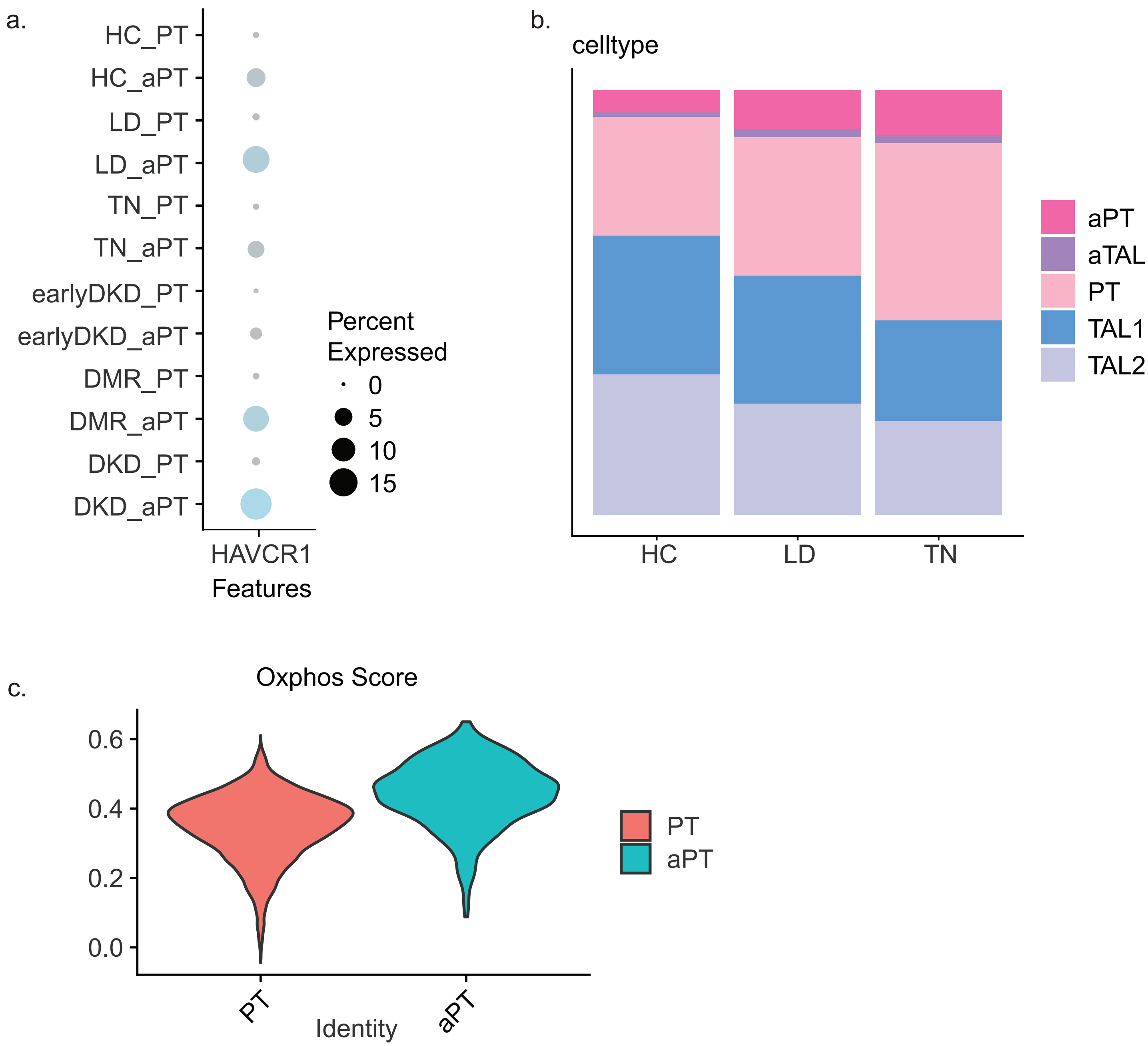

Supplementary Figure S3. Cell states. **a.** Expression levels of HAVCR1 (Hepatitis A Virus Cellular Receptor 1), also known as TIM-1 (T-cell immunoglobulin mucin domain-1) or KIM-1 (Kidney Injury Molecule-1) in proximal cell states. **b.** Proportion of cell states in proximal epithelial and thick ascending loop cells in reference groups. **c.** Oxidative phosphorylation in the PT cell states (PT and aPT) in DM-R.

Abbreviations: HC- healthy controls; LD- living kidney donor; TN- tumor nephrectomy; PT-proximal tubule, adaptive/maladaptive proximal tubule - aPT; TAL-thick ascending loop of Henle; adaptive/maladaptive thick ascending loop of Henle-aTAL.

Supplementary Figure S4

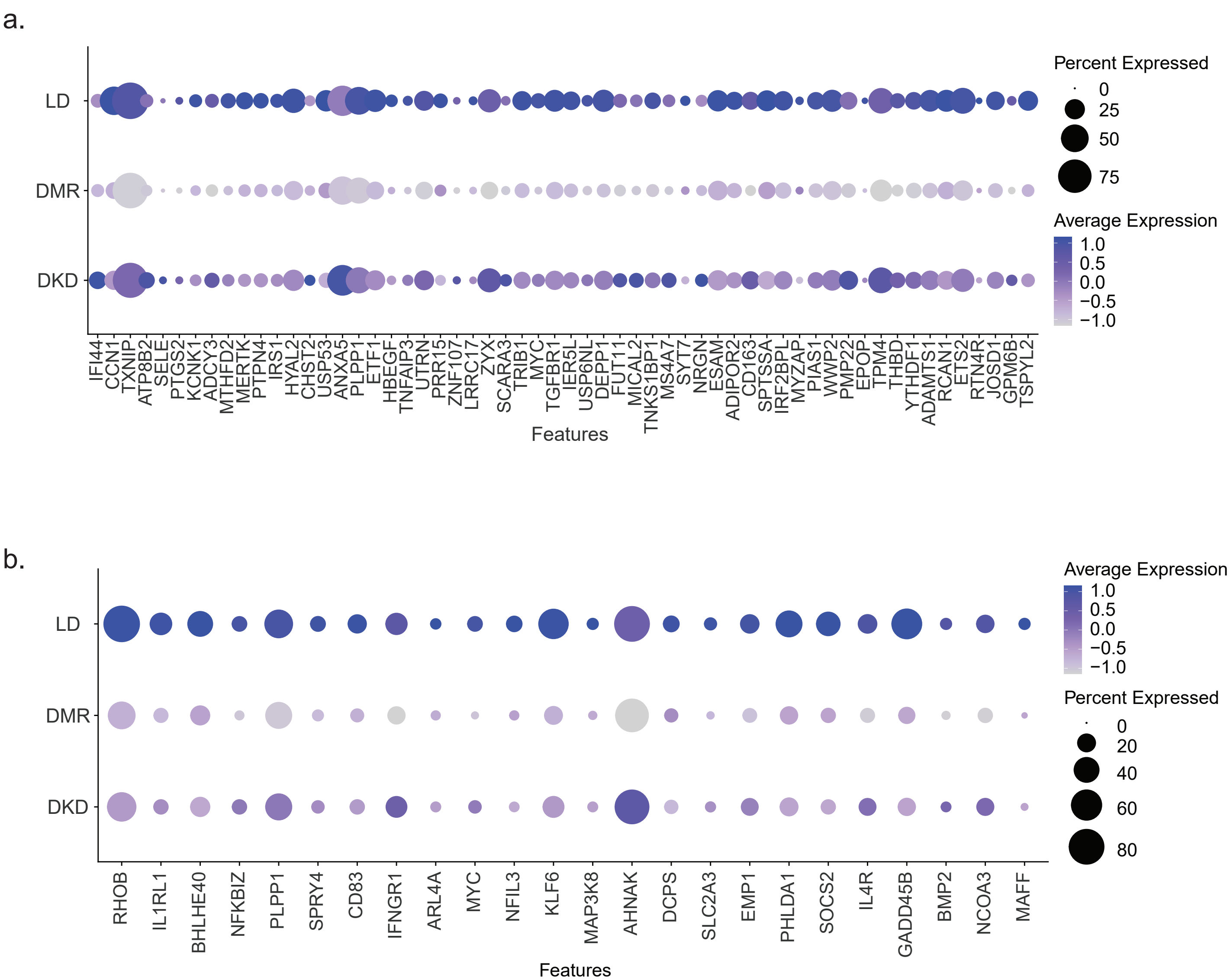

Supplementary Figure S4. TNFA signaling and IL2-STAT5 pathway genes a. Dot plot showing the expression pattern of genes involved in TNFA signaling (Hallmark pathway TNFA_SIGNALING_VIA_NFKB) in LD, DM-R and DKD. Only the genes that were significantly (p.adj < 0.05) differentially expressed in DKD versus LD are shown. **b.** Dot Plot showing the expression pattern of genes involved in IL2/STAT5 signaling (Hallmark pathway: IL2_STAT5_SIGNALING) in LD, DM-R and DKD. Only the genes that were significantly (p.adj < 0.05) differentially expressed in DM-R versus LD are shown.
Abbreviations: LD - living kidney donor; DMR - diabetes mellitus resistor; DKD - diabetic kidney disease;

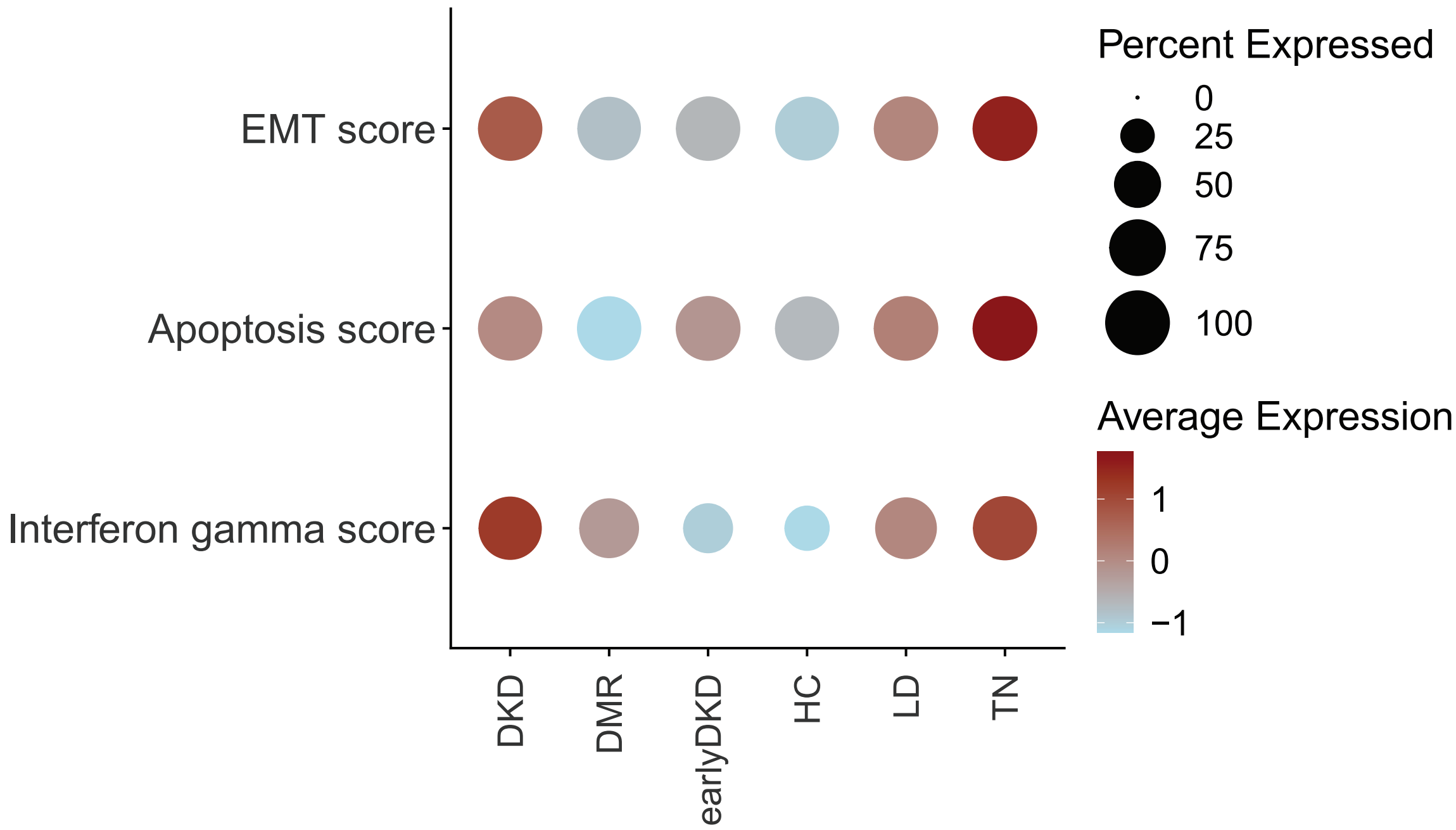

Supplementary Figure S5. Pathway scores. Dot plot showing the scores generated for the genes involved in epithelial-to-mesenchymal transition (EMT), apoptosis and interferon gamma response in the visium data of all 6 sample groups. 15 samples each in LD and DM-R, 13 samples in DKD, 5 samples in HC, 4 samples in early DKD and 2 samples in TN
Abbreviations: Healthy control HC, Living donor LD, Tumor-nephrectomy TN, Diabetes mellitus resilient DM-R, early diabetic kidney disease earlyDKD, Diabetic kidney disease DKD
